# Knowledge deficit, attitude and behavior scales association to objective measures of sun exposure and sunburn in a Danish population based sample

**DOI:** 10.1371/journal.pone.0178190

**Published:** 2017-05-25

**Authors:** Brian Køster, Jens Søndergaard, Jesper Bo Nielsen, Karl Bang Christensen, Martin Allen, Anja Olsen, Joan Bentzen

**Affiliations:** 1 Department of Prevention and Information, Danish Cancer Society, Strandboulevarden 49, Copenhagen Ø, Denmark; 2 Research Unit of General Practice, University of Southern Denmark,Odense, Denmark; 3 Section of Biostatistics, Department of Public Health, University of Copenhagen, Copenhagen, Denmark; 4 Electrical and Computer Engineering, University of Canterbury, Christchurch, New Zealand; 5 Research Centre, Danish Cancer Society, Copenhagen, Denmark; University of Colorado Denver School of Medicine, UNITED STATES

## Abstract

The objective of this study was to develop new scales measuring knowledge and attitude about UVR and sun related behavior, and to examine their association to sun related behavior objectively measured by personal dosimetry. During May-August 2013, 664 Danes wore a personal electronic UV-dosimeter for one week that measured their UVR exposure. Afterwards, they answered a questionnaire on sun-related items. We applied descriptive analysis, linear and logistic regression analysis to evaluate the associations between the questionnaire scales and objective UVR measures. Perceiving protection as routine and important were positively correlated with protective behavior. *Knowledge deficit of UV and risk of melanoma*, *perceived benefits* and *importance of protection behavior* was also correlated with use of protection. ‘*Knowledge deficit of UV and risk of melanoma* and *Perceived barrier towards sun avoidance between 12 and 15’* were both associated with increased risk of sunburn. *Attitude towards tan* was associated to both outdoor time and exposure as well as use of protection, but not to sunburn. The results regarding *Knowledge deficit of UV and risk of melanoma* associated to UVR exposure and *Perceived barrier towards sun avoidance between 12 and 15* emphasize the importance of awareness of melanoma risk and the priority of the skin cancer prevention advice. Shifting activities to outside the suns peak-hours could be an approach for structural and campaign preventive measures. Knowledge of items predicting exposure to UVR, use of protection and sunburn are important for planning of preventive interventions and melanoma research.

## Introduction

Incidence of both malignant and non-malignant skin cancer have increased for decades in large parts of the western world and especially in Caucasian populations [1]. The incidence of melanoma (world standardized incidence rate pr. 100.000) in Denmark in 2009–2013 for men and women was 21.2 and 26.2 new cases pr. 100.000 persons, respectively [2]. The main risk factor for skin cancer is exposure to ultraviolet radiation (UVR) from the sun and from artificial sources [3]. Ultraviolet radiation is typically divided in UVA (320–400 nm), UVB (280–320 nm) and UVC (200–280 nm). Only UVA and UVB reaches the surface of the earth. In addition to the carcinogenic effects [4], UVA is known to influence collagen, thereby causing wrinkles while UVB is the primary inducer of erythema. UVB has beneficial effects for humans as well. The most predominant being vitamin D production [5–7], while a range of less well exploited mechanisms also exists [8,9]. It has been suggested that the majority of all skin cancers could be prevented by behavior changes [10,11]. Exposure to artificial sources of radiation could easily be prevented by structural prevention if supported politically [12], however reducing exposure to natural UVR from the sun is dependent on e.g. skin cancer prevention campaigns to influence population behavior. Campaigns aimed at changing attitudes and behavior towards UV exposure in the general population have been launched in several countries [13–16]. Nevertheless, 22% of the Danes (aged 15–64) reported to be sunburned in the summer of 2014 in the annual national population-based survey of the Danish Cancer Society [17]. Sunburn is until now the most common used proxy measure for personal exposure to UVR [6].

The effects of these initiatives and campaigns are generally evaluated by distribution of questionnaires [18], which are suitable for collecting knowledge from representative population-based samples. Evaluation can involve direct and indirect measures. Measures of the behavior are direct measures, while indirect measures include e.g. knowledge of risk factors, knowledge of protection, attitude towards tanning behavior, and beliefs of benefits and efficiency. However, bias (recall, selection, social desirability answers) can potentially limit the reliability of conclusions drawn based on questionnaire data and it is thus essential that questionnaires are evaluated for validity and reliability [19–21]. To secure deep and complete coverage of the areas of interest, questionnaires can be based on scales. The benefit of constructing a scale is that the complete concept is covered, and thereby a scale gives a more complete image compared to the partial components [22]. Scale validation done according to item response theory using the Rasch-model is regarded the current choice of validation [23–25]. Previously other models were used [26–28]. Concept validation using the Rasch model was not previously applied in the area of skin cancer prevention.

Objective reference measurements of personal ultraviolet radiation will improve the quality of evaluation. Previous validation models only used self-reported subjective collected reference data as e.g. sun burn as a proxy measure for the carcinogenic radiation. Better knowledge for preventive efforts would be provided by the use of objectively collected data from measurements of personal exposure to UVR. Questionnaire behavioral data collection was recently validated in a small Australian study [29] and a larger Danish population based study [30]. In intervention evaluations aimed at decreasing skin cancer incidence, one or more of the parameters knowledge deficit, attitude, behavior and sunburn were used as objectives [18,31–37]. However, factors explaining the behavior as e.g. attitude and knowledge deficit, was not previously associated to objective measures of the UV-exposure.

Current skin cancer prevention campaigns are based on various theoretical models e.g. Health belief model, or Theory of planned behavior according to results from known behavioral research [26,38]. The Danish Sun Safety Campaign was primarily based on Theory of planned behavior (TpB) [38,39]. The primary short-term measures of the campaign involves the behavior leading to the event, i.e. duration of exposure to UVR and nature of the exposure, e.g. intentional sunbathing. A change in the behavior of a population may however not be implemented overnight by a campaign [40]. A behavior change needs to be preceded by an increase in awareness, a change in normative beliefs or other model components.

The objective of this study was to identify and examine new and already known components related to UVR exposure behavior. We developed new scales measuring knowledge deficit and attitude about UVR and sun related behavior and we examined a number new or previously developed scales association to sun related behavior objectively measured by personal dosimetry.

## Results

**Fig 1:**
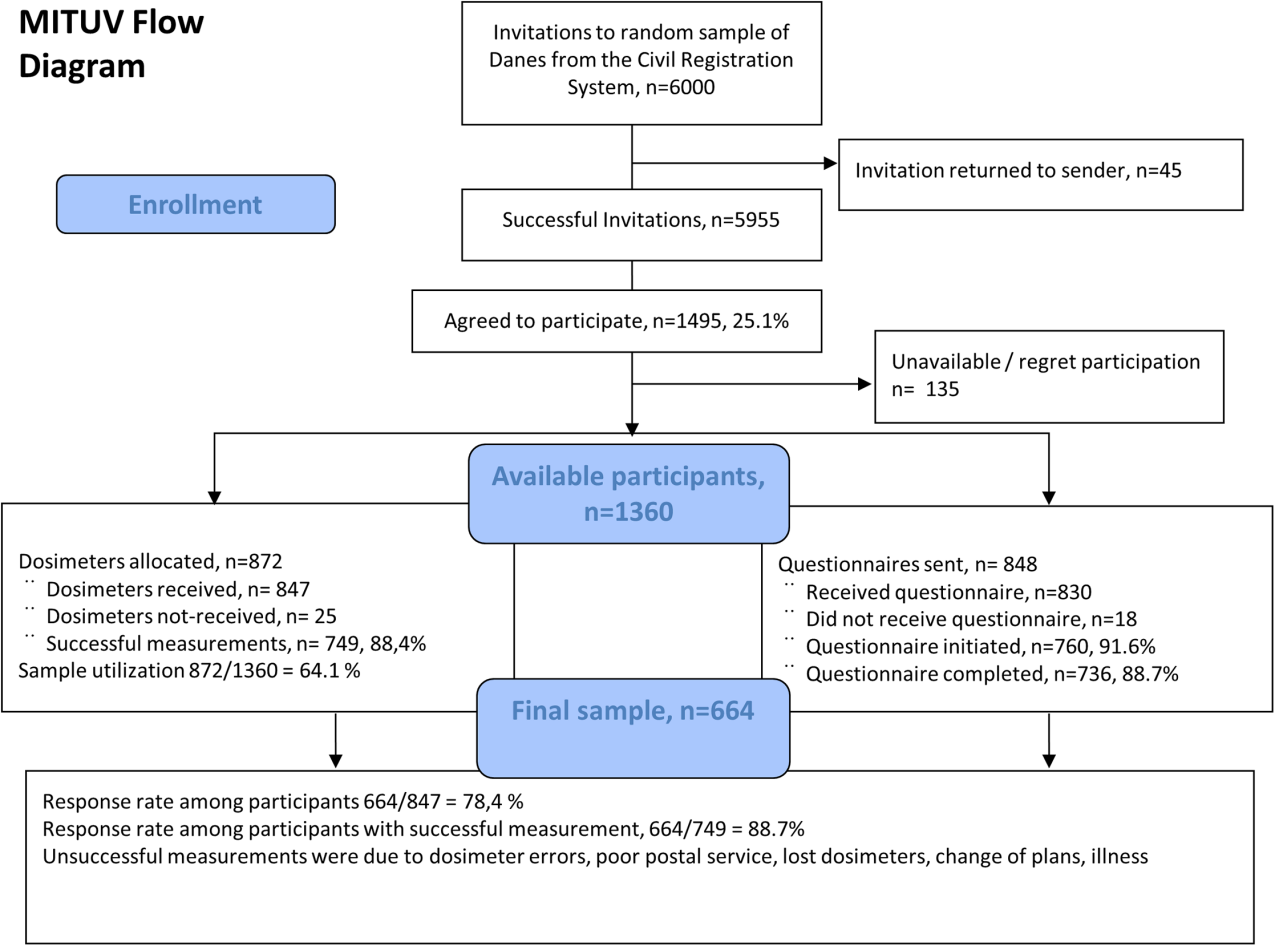
In the figure is shown the flow of participants in the project including participation and completion of uv-measurement and questionnaire.

Fig 1 shows the flow of the study. Six thousand persons were invited and of those 25% signed up for participation. We collected data from 749 successful dosimeter measurements and we received 736 completed questionnaires and for 664 persons we have complete data for both dosimetry and questionnaire with a response rate of 89%.

Table 1 shows the knowledge deficit and attitude scales examined. It also shows the scale scores distribution by demographic characteristics. In addition, in S1 Table the items of the scales is shown, including means, rest score correlation, item-item correlation range as well as item fit statistics. We identified 4 knowledge deficit scales: *Uv and risk of melanoma* is composed of 6 items on risk of melanoma in relation to sun beds, sunburn as adult, travelling to sunny destinations, staying in the sun between 12pm and 3 pm, sunbathing and outdoor work. *UV exposure/penetration* is composed of 5 items on shade, not sunbathing, water, clouds and rain. *UV types and cancer* is composed of 3 items on UVA, UVB and UVC. *UV and Vitamin D synthesis* is composed of 6 items on exposure in the shade, exposure between 12pm and 3 pm, sunbathing, sunscreen, correct and incorrect exposure time. We identified 5 scales about beliefs of MM and Skin examination including *Perceived severity of Melanoma* (belief that malignant melanoma is easy curable, MM can have serious consequences, getting MM will be a large health risk for me), *Worry about Melanoma* (probability of developing skin cancer worries me, hearing of persons with skin cancer makes me think I can get it, getting skin cancer would be terrible), *Skin examination self efficacy* (frequency of …self examination, examination by family member, examination by health professional), *Perceived efficiency of skin examination* (examination of health professional can identify skin cancer not yet developed, Regular skin examination will make me less worried about my health, Regular skin examination will help me to a long life, Regular self examination of my skin will help me identify skin changes before they are serious, self examination of my skin for changes makes me feel in control of my health), *Perceived barriers of skin examination* (regular consultancy of physician for skin examination too expensive and time consuming, uncomfortable and embarrassed about a physician examining my skin, I worry when a physician examines my skin for changes, I worry when I examine my skin for changes, I am not very good at examining my skin for changes). We identified 8 scales in relation to protection *Perceived importance of protection against outdoor exposure* (3 items clothing, avoid sun between 12pm and 3 pm and hat), *Perceived benefits of protection behavior* (8 items sunscreen against cancer, sunburn and ageing, clothing against cancer and ageing, avoid sun between 12pm and 3 pm against ageing, shade against cancer and hat against burn), *Perceived protection as routine* (4 items sunscreen, clothing, avoid sun between 12pm and 3 pm and hat), *Perceived protection as barrier against tan* (4 items sunscreen, clothing, avoid sun between 12pm and 3 pm and hat), and 4 scales on *perceived barriers for using the protection methods*:*sunscreen* (3 items difficult, expensive, disturbing), *clothing* (5 items difficult, inconvenient, embarrassing, uncomfortable, disturbing), *avoid sun between 12pm and 3 pm* (4 items difficult, inconvenient, suit well, disturbing) and *hat* (3 items embarrassing, uncomfortable, disturbing).

**Table 1:** Distribution of demographic characteristics and scale scores in a cross-sectional sample of 664 Danes.

Characteristic (%)	Total	%	Knowledge deficit UV risk of melanoma (0–18)	Knowledge deficit UV penetration (0–15)	Knowledge deficit UV types (0–9)	Knowledge deficit UV and vitamin d (0–18)	Severity of Melanoma (0–12)	Worry about Melanoma (0–12)	Skin examination self efficacy (0–6)	Perceived efficiency of skin examination (0–20)	Perceived barriers of Skin examination (0–20)	
Total (n = 664)												
Total	664	100	4.4 (3.3)	6.0 (3.0)	4.6 (1.9)	8.2 (3.3)	2.8 (2.0)	3.5 (2.1)	4.3 (1.4)	12.6 (3.2)	8.1 (3.4)	
Gender			*p<0*.*001*	*p<0*.*001*	*p = 0*.*065*	*p = 0*.*020*	*p<0*.*001*	*p<0*.*001*	*p = 0*.*002*	*p = 0*.*09*	*p = 0*.*80*	
Male	251	38	5.2 (3.4)	6.5 (3.2)	4.7 (1.9)	8.6 (3.4)	3.2 (2.0)	4.1 (2.1)	4.5 (1.4)	12.4 (3.0)	8.1 (3.4)	
Female	413	62	3.9 (3.2)	5.7 (2.9)	4.4 (1.9)	8.0 (3.3)	2.6 (2.0)	3.2 (2.0)	4.2 (1.4)	12.8 (3.4)	8.2 (3.3)	
Agegroup			*p<0*.*001*	*p<0*.*001*	*p = 0*.*006*	*p<0*.*001*	*p = 0*.*23*	*p = 0*.*005*	*p = 0*.*007*	*p = 0*.*59*	*p<0*.*001*)	
15–24	100	15	4.8 (3.1)	6.3 (4.5)	4.5 (1.7)	8.8 (3.0)	2.9 (1.8)	3.4 (2.0)	4.7 (1.4)	12.4 (2.7)	9.4 (3.0)	
25–34	104	16	3.2 (2.7)	4.5 (2.4)	4.0 (2.1)	7.0 (3.4)	2.7 (2.0)	3.1 (2.1)	4.1 (1.5)	12.9 (3.2)	8.5 (3.5)	
35–44	118	18	3.7 (3.1)	5.4 (2.9)	4.4 (2.0)	7.8 (3.1)	2.5 (2.0)	3.2 (1.9)	4.6 (1.2)	12.8 (3.2)	9.0 (3.1)	
45–54	132	20	4.7 (3.5)	6.2 (3.3)	4.6 (1.9)	8.1 (3.3)	2.7 (2.1)	3.7 (2.2)	4.2 (1.4)	12.9 (3.5)	7.2 (3.2)	
55–65	210	31	5.0 (3.6)	6.9 (3.0)	4.9 (1.7)	8.7 (3.4)	3.0 (2.0)	3.9 (2.1)	4.2 (1.4)	12.4 (3.3)	7.5 (3.4)	
Skintype			*p = 0*.*001*	*p = 0*.*13*	*p = 0*.*14*	*p = 0*.*18*	*p = 0*.*67*	*p = 0*.*005*	*p = 0*.*36*	*p = 0*.*24*	*p = 0*.*03*	
I	54	8	3.9 (2.5)	5.2 (2.6)	4.4 (1.8)	7.9 (3.1)	2.8 (1.9)	3.6 (2.2)	4.1 (1.5)	13.4 (2.6)	8.5 (3.1)	
II	383	58	4.1 (3.1)	6.0 (3.0)	4.5 (2.0)	8.0 (3.3)	2.7 (1.9)	3.3 (2.0)	4.3 (1.4)	12.6 (3.3)	8.4 (3.4)	
III/ IV	227	35	5.0 (3.7)	6.2 (3.2)	4.8 (1.7)	8.5 (3.3)	2.9 (2.1)	3.9 (2.2)	4.4 (1.3)	12.5 (3.2)	7.7 (3.3)	
Region			*p = 0*.*019*	*p = 0*.*006*	*p = 0*.*021*	*p = 0*.*13*	*p = 0*.*31*	*p = 0*.*41*	*p = 0*.*04*	*p = 0*.*07*	*p = 0*.*36*	
Capital	187	28	3.8 (3.1)	5.3 (2.7)	4.2 (2.1)	7.7 (3.4)	2.6 (2.1)	3.4 (2.0)	4.2 (1.4)	13.1 (3.2)	7.9 (3.4)	
Zealand	103	16	4.3 (3.3)	6.3 (3.4)	4.6 (1.9)	8.2 (3.3)	2.7 (1.9)	3.4 (1.9)	4.3 (1.4)	13.0 (3.3)	8.1 (3.2)	
Northern Jutland	68	10	4.3 (3.3)	6.5 (3.4)	4.7 (1.8)	8.4 (3.5)	2.9 (2.0)	3.5 (2.4)	4.1 (1.5)	12.2 (2.9)	8.0 (3.8)	
Central Jutland	167	25	5.0 (3.4)	6.1 (3.0)	4.8 (1.7)	8.3 (3.2)	2.9 (1.9)	3.8 (2.0)	4.6 (1.3)	12.2 (3.2)	8.6 (3.5)	
Southern Denmark	139	21	4.6 (3.5)	6.3 (2.9)	4.7 (1.8)	8.6 (3.2)	3.1 (2.1)	3.6 (2.3)	4.3 (1.4)	12.5 (3.3)	8.1 (3.0)	
Education			*p = 0*.*003*	*p<0*.*001*	*p = 0*.*008*	*p<0*.*001*	*p<0*.*001*	*p = 0*.*10*	*p = 0*.*05*	*p = 0*.*86*	*p = 0*.*008*	
Primary school	117	18	5.0 (3.8)	7.5 (3.8)	5.0 (1.6)	9.5 (3.0)	3.5 (1.9)	3.6 (2.2)	4.5 (1.4)	12.7 (3.1)	8.6 (3.2)	
Secondary school	90	14	4.8 (3.1)	5.9 (3.1)	4.5 (1.8)	7.9 (3.2)	2.8 (1.9)	3.2 (1.8)	4.5 (1.3)	12.8 (2.9)	9.1 (3.3)	
Vocational	91	14	4.8 (3.6)	6.5 (3.0)	4.9 (1.6)	8.8 (3.4)	3.4 (2.1)	3.8 (1.9)	4.4 (1.3)	12.5 (3.1)	8.0 (3.4)	
Higher education (<2y)	67	10	4.6 (3.3)	6.0 (2.7)	4.7 (1.7)	8.2 (3.0)	2.9 (1.8)	3.7 (2.4)	4.2 (1.3)	12.6 (3.1)	7.2 (3.7)	
Higher education (2–4½y)	213	32	4.0 (3.2)	5.4 (2.9)	4.4 (2.0)	7.8 (3.4)	2.5 (2.0)	3.7 (2.2)	4.1 (1.4)	12.5 (3.6)	7.8 (3.2)	
Higher education (>4½y)	80	12	3.3 (2.4)	4.6 (2.8)	3.8 (2.3)	7.0 (3.1)	1.9 (1.8)	3.0 (1.7)	4.4 (1.3)	12.8 (3.2)	8.1 (3.5)	
Own or family related melanoma			*p = 0*.*005*	*p = 0*.*05*	*p = 0*.*15*	*p = 0*.*31*	*p = 0*.*04*	*p = 0*.*008*	*p = 0*.*002*	*p<0*.*001*)	*p = 0*.*51*	
Yes	147	22	3.7 (3.2)	5.6 (3.3)	4.4 (1.9)	7.9 (3.2)	2.5 (1.9)	3.1 (2.4)	4.0 (1.5)	13.5 (3.3)	8.0 (3.4)	
No	517	78	4.6 (3.3)	6.1 (3.0)	4.6 (1.9)	8.3 (3.3)	2.9 (2.0)	3.6 (2.0)	4.4 (1.3)	12.4 (3.2)	8.2 (3.3)	
Weather			*p = 0*.*008*	*p = 0*.*70*	*p = 0*.*022*	*p = 0*.*45*	*p = 0*.*05*	*p = 0*.*28*	*p = 0*.*31*	*p = 0*.*18*	*p = 0*.*67*	
1 (Most of the time sunny)	191	29	4.4 (3.4)	5.8 (3.0)	4.6 (2.0)	8.0 (3.3)	2.6 (2.1)	3.4 (2.0)	4.3 (1.4)	12.7 (3.2)	7.9 (3.4)	
2	176	27	4.5 (3.2)	6.2 (2.9)	4.9 (1.6)	8.4 (3.1)	3.1 (1.8)	3.7 (2.0)	4.4 (1.4)	12.8 (3.0)	8.3 (3.1)	
3	152	23	4.6 (3.3)	6.1 (3.0)	4.2 (2.0)	8.1 (3.4)	2.7 (2.0)	3.7 (2.0)	4.2 (1.5)	12.4 (3.6)	8.0 (3.6)	
4	83	12	3.8 (3.4)	6.1 (3.5)	4.4 (1.9)	8.5 (3.4)	3.2 (2.2)	3.2 (2.0)	4.4 (1.3)	13.3 (3.4)	8.4 (3.4)	
5 (Most of the time clouded)	61	9	4.3 (3.3)	6.0 (2.6)	4.5 (2.1)	8.0 (3.5)	2.5 (1.9)	3.6 (2.2)	4.6 (1.2)	12.0 (2.9)	8.5 (3.4)	
Characteristic **(%)**	Total	%	Perceived importance of protection behavior (0–12)	Perceived benefits of protection behavior (0–32)	Protection routine (0–16)	Protection barrier color (0–16)	Perceived barriers sunscreen (0–12)	Perceived barriers clothing (0–20)	Perceived barriers sun avoidance (0–16)	Perceived barriers hat (0–12)	Attitude tanning own (0–28)	**Use of Protection** (0–21)
Total (n = 664)												
Total	664	100	6.0 (2.4)	23.0 (5.0)	9.6 (2.7)	6.3 (3.2)	3.7 (2.3)	8.9 (4.0)	8.6 (3.4)	5.2 (3.0)	17.0 (4.3)	5.4 (3.7)
Gender			*p = 0*.*014*	*p = 0*.*008*	*p = 0*.*85*	*p = 0*.*064*	*p<0*.*001*	*p = 0*.*33*	*p = 0*.*21*	*p<0*.*001*	*p<0*.*001*	*p = 0*.*030*
Male	251	38	5.7 (2.5)	22.4 (5.0)	9.6 (2.8)	6.0 (3.4)	4.5 (2.3)	8.7 (4.0)	8.8 (3.4)	4.4 (2.9)	15.9 (4.4)	5.0 (3.4)
Female	413	62	6.2 (2.4)	23.4 (4.9)	9.6 (2.7)	6.5 (3.0)	3.2 (2.2)	9.0 (4.0)	8.5 (3.4)	5.7 (2.9)	17.7 (4.0)	5.6 (3.9)
Agegroup			*p<0*.*001*	*p<0*.*001*	*p<0*.*001*	*p<0*.*001*	*p = 0*.*07*	*p = 0*.*05*	*p<0*.*001*	*p = 0*.*01*	*p<0*.*001*	*p = 0*.*13*
15–24	100	15	4.3 (2.4)	21.6 (4.8)	10.7 (2.4)	7.8 (3.5)	3.8 (1.7)	9.8 (3.9)	9.4 (3.1)	6.1 (2.7)	17.8 (4.3)	4.8 (3.8)
25–34	104	16	6.0 (2.2)	25.4 (4.8)	9.9 (2.3)	5.8 (3.1)	3.3 (2.3)	8.7 (3.6)	9.1 (3.7)	5.2 (3.1)	17.5 (4.2)	6.0 (3.8)
35–44	118	18	6.0 (2.5)	23.9 (4.2)	9.9 (6.4)	6.4 (3.3)	3.6 (2.4)	9.1 (4.4)	9.3 (3.1)	5.3 (3.2)	17.7 (3.9)	4.9 (3.2)
45–54	132	20	6.3 (2.4)	23.3 (5.2)	9.4 (2.7)	6.4 (3.3)	3.6 (2.2)	9.1 (4.0)	8.3 (3.2)	5.4 (2.9)	17.6 (3.8)	5.3 (3.8)
55–65	210	31	6.5 (2.2)	21.8 (4.8)	8.9 (2.9)	5.9 (2.9)	4.0 (2.5)	8.3 (3.9)	7.7 (3.4)	4.7 (3.0)	15.6 (4.5)	5.6 (3.9)
Skintype			*p = 0*.*12*	*p = 0*.*005*	*p = 0*.*10*	*p = 0*.*040*	*p = 0*.*018*	*p = 0*.*33*	*p = 0*.*04*	*p = 0*.*43*	*p = 0*.*17*	*p<0*.*001*
I	54	8	6.2 (2.2)	24.7 (4.6)	9.0 (2.8)	5.3 (3.6)	3.0 (2.1)	8.5 (3.8)	7.7 (3.8)	5.7 (3.1)	16.0 (4.7)	7.5 (4.1)
II	383	58	6.1 (2.4)	23.2 (4.7)	9.5 (2.7)	6.4 (3.1)	3.7 (2.3)	8.8 (4.0)	8.5 (3.3)	5.1 (3.0)	17.0 (4.0)	5.6 (3.7)
III/ IV	227	35	5.7 (2.6)	22.3 (5.3)	9.9 (2.7)	6.5 (3.4)	4.0 (2.4)	9.2 (4.0)	8.9 (3.4)	5.3 (3.1)	17.2 (4.5)	4.4 (3.5)
Region			*p = 0*.*24*	*p = 0*.*004*	*p = 0*.*70*	*p = 0*.*005*	*p = 0*.*53*	*p = 0*.*19*	*p = 0*.*65*	*p = 0*.*002*	*p = 0*.*51*	*p = 0*.*25*
Capital	187	28	6.1 (2.4)	24.2 (5.3)	9.5 (2.5)	5.8 (3.2)	3.6 (2.3)	8.7 (4.0)	8.7 (3.4)	4.7 (2.7)	17.0 (4.0)	5.8 (3.6)
Zealand	103	16	6.3 (2.4)	22.5 (5.3)	9.4 (2.8)	6.4 (2.9)	3.8 (2.4)	9.0 (4.0)	8.6 (3.4)	5.1 (3.1)	17.0 (4.0)	5.6 (4.1)
Northern Jutland	68	10	5.7 (2.3)	22.9 (4.0)	10.0 (2.8)	5.7 (3.7)	3.4 (2.4)	8.1 (4.0)	8.7 (3.6)	4.6 (3.1)	16.4 (4.3)	5.4 (3.9)
Central Jutland	167	25	5.7 (2.5)	22.2 (4.9)	9.7 (2.6)	6.9 (3.3)	3.8 (2.3)	9.4 (4.1)	8.8 (3.4)	5.9 (2.9)	17.4 (4.2)	5.0 (3.6)
Southern Denmark	139	21	6.0 (2.5)	22.8 (4.6)	9.5 (3.0)	5.5 (3.2)	3.9 (2.2)	8.8 (3.8)	8.2 (3.3)	5.5 (3.2)	16.8 (4.8)	5.1 (3.7)
Education			*p = 0*.*20*	*p<0*.*001*	*p = 0*.*06*	*p = 0*.*045*	*p = 0*.*17*	*p = 0*.*12*	*p = 0*.*73*	*p = 0*.*09*	*p = 0*.*30*	*p = 0*.*39*
Primary school	117	18	5.8 (2.8)	21.5 (4.5)	9.2 (2.7)	7.0 (2.9)	4.0 (2.2)	9.8 (3.8)	8.6 (3.1)	5.9 (2.8)	16.2 (4.8)	5.0 (3.9)
Secondary school	90	14	5.4 (2.5)	22.7 (4.3)	10.0 (2.7)	6.7 (3.4)	3.9 (2.2)	8.9 (4.2)	8.8 (3.4)	5.4 (3.0)	17.0 (4.0)	5.5 (3.8)
Vocational	91	14	5.9 (2.6)	21.9 (4.9)	9.4 (2.4)	6.3 (3.3)	4.1 (2.5)	9.1 (3.8)	8.7 (3.4)	5.0 (3.7)	16.5 (4.4)	5.0 (3.7)
Higher education (<2y)	67	10	6.4 (2.0)	22.9 (4.8)	9.3 (2.8)	6.7 (3.4)	3.5 (2.4)	8.3 (4.2)	8.3 (3.7)	5.1 (3.0)	17.0 (4.2)	5.3 (3.6)
Higher education (2–4½y)	213	32	6.1 (2.3)	23.5 (5.2)	9.9 (2.7)	6.0 (3.2)	3.5 (2.3)	8.7 (4.0)	8.5 (3.6)	5.4 (3.8)	17.5 (4.5)	5.4 (3.8)
Higher education (>4½y)	80	12	6.1 (2.3)	25.5 (4.8)	9.7 (3.1)	5.6 (3.3)	3.5 (2.4)	8.3 (4.0)	8.8 (3.3)	4.5 (2.5)	17.4 (4.1)	6.1 (3.4)
Own or family related melanoma			*p = 0*.*77*	*p = 0*.*009*	*p = 0*.*41*	*p = 0*.*91*	*p = 0*.*20*	*p = 0*.*78*	*p = 0*.*52*	*p = 0*.*68*	*p = 0*.*02*	*p = 0*.*027*
Yes	147	22	6.0 (2.4)	24.2 (4.8)	9.4 (2.7)	6.3 (3.2)	3.5 (2.4)	9.0 (4.0)	8.7 (3.5)	5.3 (3.4)	17.7 (3.9)	6.0 (4.1)
No	517	78	6.0 (2.5)	22.7 (5.0)	9.6 (2.7)	6.4 (3.2)	3.8 (2.3)	8.9 (4.0)	8.5 (3.4)	5.2 (2.9)	16.8 (4.4)	5.2 (3.6)
Weather			*p = 0*.*93*	*p = 0*.*91*	*p = 0*.*61*	*p = 0*.*035*	*p = 0*.*69*	*p = 0*.*19*	*p = 0*.*75*	*p = 0*.*09*	*p = 0*.*90*	*p = 0*.*27*
1 (Most of the time sunny)	191	29	6.0 (2.3)	23.1 (5.0)	9.7 (2.8)	6.0 (3.4)	3.6 (2.4)	9.1 (4.2)	8.8 (3.3)	5.0 (3.2)	17.0 (4.2)	5.1 (3.2)
2	176	27	5.8 (2.3)	23.0 (4.8)	9.7 (2.6)	6.3 (3.1)	3.8 (2.2)	9.0 (3.7)	8.5 (3.5)	5.4 (2.7)	16.7 (4.1)	5.3 (3.6)
3	152	23	6.0 (2.5)	22.9 (5.0)	9.5 (2.8)	6.1 (3.0)	3.8 (2.5)	8.2 (3.9)	8.6 (3.9)	4.9 (3.1)	16.8 (4.5)	5.8 (4.0)
4	83	12	6.1 (2.7)	22.9 (5.3)	9.2 (2.9)	7.1 (3.4)	3.9 (2.1)	9.6 (4.2)	8.1 (3.0)	5.8 (4.4)	17.2 (3.9)	5.8 (4.4)
5 (Most of the time clouded)	61	9	6.1 (2.6)	23.3 (4.7)	9.9 (2.5)	7.1 (3.2)	3.4 (2.2)	8.6 (3.8)	8.7 (3.4)	5.2 (3.1)	17.4 (4.9)	4.7 (4.1)

Values are mean (SD). Increasing values of score indicates a higher degree of the subject in question. p-values are from t-tests or anova as appropriate

We also identified a scale of *Attitude towards own tanning* (Tan is healthy, tan makes me look healthy, Tan makes me look better, tan makes me feel comfortable, Do not think about tan much, does not like to lay in the sun, does not like to be completely pale. Finally, we show the *protection scale* (7 items, sunscreen SPF15+, long sleeves, long trouser/skirt, cap, wide brimmed hat, shade, stay inside between 12pm- 15pm), which was previously described [30].

In general, women scored lower than men did on the knowledge deficit scales indicating a higher knowledge. People aged 25–44 also scored lower, as well as people with higher education and family related skin cancer. For *UV and risk of melanoma* people with skin types, I or II scored lower than Skin type III, while there was no differences between skin type and the other knowledge deficit scales.

For *perceived importance of protection* and for *perceived benefits of protection* behavior women scored higher than men indicating higher agreement with importance and benefits. In addition, the youngest age group (15–24 years) scored lower in all three scales. For perceived benefits, additionally the elderly (55–65) scored lower and there was a lower score with darker skin type.

*Perceived protection as routine* was lower with increasing age indicating better sun protection routines with increasing age. *Perceived protection as barrier against tan* was higher among women, age group 15–24 years and skin type I indicating a larger barrier in these groups. For *perceived barriers for using the protection methods* men had a larger barrier for using sunscreen, while women had a larger barrier for wearing a hat. Age group 55–65 had a larger barrier against sunscreen, age group 15–24 had a larger barrier against clothing and against wearing a hat and regarding sun avoidance between 12 pm and 3 pm the barrier decreased with age. Barriers against sunscreen and against sun avoidance between 12 pm and 3 pm increased with increasing skin type.

Women, decreasing age and skin type I had a more positive *Attitude towards own tanning* and *Attitude towards social group tanning*. Men have a lower *General risk perception*, *a lower Perceived severity of Melanoma and worries less about MM*. People with increasing length of education considers MM more severe.

*Skin examination self-efficacy* is lowest among men, age group 15–24 and people with shorter education. The only differences in the *Perceived efficiency of skin examination* is among people with a family related skin cancer diagnosis. *Barriers against skin examination* are higher in the youngest half of the sample, the shorter length of education and higher in skin type I and II relatively to skin type III.

In Table 2, we show the scale-scale correlations between selected scales that are potential predictors of protection behavior. The scales that showed the strongest positive correlations with the *protection behavior scale* are *perceived protection as routine* and *perceived importance of protection*, while the strongest negative correlations with the *protection behavior scale* are *Attitude towards tan* and *perceived barrier towards sun avoidance*. *Knowledge deficit of UV as MM risk* was less strongly, but significantly correlated to the *protection behavior scale*. The three other knowledge deficit scales (*UV penetration*, *UV types and UV and vitamin d*) were significantly correlated (0.22–0.33, *p<0*.*001*) with *Knowledge deficit of UV as MM risk*, however they were not significantly correlated to the *protection behavior scale*.

**Table 2:** Correlation of protection behavior scale and protection attitude and knowledge deficit scales.

Scale correlation	Perceived importance of protection	Perceived benefits of protection behavior	Protection is part of routine	Protection is a barrier against tan/color	Perceived barriers sunscreen	Perceived barriers clothing	Perceived barriers sun avoidance	Perceived barriers hat	Knowledge deficit of risk	Skin Examination Efficacy	Attitude Tanning	Use of Protection
Total (n = 664)												
Perceived importance of protection	—											
Perceived benefits of protection behavior	0.24***	—										
Protection routine	0.45***	0.01	—									
Protection color	-0.25***	-0.13**	-0.22**	—								
Perceived barriers sunscreen	-0.07	-0.13***	-0.11*	0.24***	—							
Perceived barriers clothing	0.33***	-0.05	-0.16***	0.34***	0.23***	—						
Perceived barriers sun avoidance	-0.29***	-0.04	-0.35***	0.21***	0.19***	0.30***	—					
Perceived barriers hat	-0.21***	-0.03	-0.20***	0.34***	0.15***	0.38***	0.15***	—				
Knowledge deficit of risk	0.25***	0.39***	0.02	-0.26***	-0.18***	-0.02	-0.07	-0.07	—			
Skin Examination Efficacy	0.23***	0.06	0.18***	-0.08*	-0.09*	-0.08*	-0.11**	-0.13**	0.13***	—		
Attitude Tanning	-0.30***	-0.09*	-0.41***	0.58***	0.01	0.24***	0.23***	0.17	0.00	-0.07	—	
**Protection score**	0.37***	0.15***	0.43***	-0.19***	-0.08**	-0.18***	-0.24***	-0.14***	0.11**	0.26***	-0.30***	**—**

Spearman correlation coefficients was applied. Significance levels are indicated by

*<0.05

**<0.01

***<0.001

We examined the association of the developed scales and the objectively measured behavior. We examined both association to outdoor exposure time and received carcinogenic UVR.

In Table 3 is shown the final models of scales predicting outdoor exposure time, outdoor radiation measured by dosimetry and protection behavior as measured by the protection scale. All scales were analyzed, however only scales with significant associations are shown. *Knowledge deficit of UV and risk of melanoma* was significantly associated to both exposure time and to standard erythemal dose (SED), but not the *protection behavior scale*. *Attitude towards tan* was contributing significantly to all three models, while *perceived barrier towards sun avoidance 12–15 was only* associated to exposure time. *Perceived protection as routine*, *Skin examination self-efficacy and Perceived protection as barrier against tan* were included as explanatory variables in both the SED and the protection behavior model. *Perceived importance of protection*, *Perceived benefits of protection behavior* and *Perceived barrier for using clothing as protection were all included to the protection scale model only*. The combined effects of exposure and lack of protection may lead to sunburn.

**Table 3:** Linear regression models of outdoor exposure time, UV-exposure received in SED and the protection scale respectively.

Characteristic	Exposure time Model	SED Model	Protection scale model
n = 664	R^2^ = 0.36, p< 0.001	R^2^ = 0.32, p< 0.001	R^2^ = 0.34, p< 0.001
	F-value (*p-value*)	F-value (*p-value*)	F-value (*p-value*)
*Knowledge UV risk of melanoma*	8.9 (*p = 0*.*003*)	3.7 (*p = 0*.*057)*	N.A.
*Attitude toward tanned look*	6.1 (*p = 0*.*01*)	10.2 (*p = 0*.*002*)	9.8 (*p = 0*.*002)*
*Perceived barrier towards avoiding sun 12–15*	11.6 (*p < 0*.*001*)	N.A.	N.A.
*Routine*	N.A.	5.8 (*p = 0*.*016*)	62.9 (*p < 0*.*001)*
*Perceived barrier not tanning*	N.A.	4.4 (*p = 0*.*036*)	4.0 (*p = 0*.*047)*
*Skin examination self efficacy*	N.A.	4.7 (*p = 0*.*031*)	7.9 (*p = 0*.*005)*
*Perceived importance of protection behavior*	N.A.	N.A.	8.4 (*p = 0*.*004)*
*Perceived benefits of protection behavior*	N.A.	N.A.	8.0 (*p = 0*.*005)*
*Perceived barriers clothing*	N.A.	N.A.	4.7 (*p = 0*.*031)*
Week of participation	5.8 (*p < 0*.*001*)	4.8 (*p < 0*.*001*)	1.9 (*p = 0*.*021)*
Age	10.6 (*p < 0*.*001*)	28.3 (*p < 0*.*001*)	1.9 (*p = 0*.*11)*
Weather	8.2 (*p < 0*.*001*)	4.9 (*p < 0*.*001*)	0.6 (*p = 0*.*72)*
Skintype	0.9 (*p = 0*.*46*)	2.3 (*p = 0*.*07*)	5.9 (*p < 0*.*001)*
Gender	0.3 (*p = 0*.*56*)	0.0 (*p = 0*.*92*)	5.6 (*p = 0*.*019)*
Education	1.3 (*p = 0*.*26*)	1.5 (*p = 0*.*19*)	1.1 (*p = 0*.*37)*

All scales were examined, however only scales with significant associations in any of the models are included in the table.

In Table 4, we have examined the association of the attitudinal and knowledge deficit scales and sunburn. Increased knowledge deficit of UV and the risk of melanoma was associated to an increased risk of sunburn. *Perceived barrier towards avoiding the sun between 12–15* was the scale with the strongest association to sunburn. *Attitude towards tan* was not included in the sunburn model as it was not associated with sunburn.

**Table 4:** Logistic regression models of sunburn and background variables, knowledge deficit, attitude and behavior scales.

Characteristic	Unadjusted	Adjusted^1^
n = 664		
*Knowledge deficit UV risk of melanoma*	*p = 0*.*15* 1.04 (1.00–1.09)	*p = 0*.*041* 1.06 (1.00–1.13)
*Attitude toward tanned look*	*p = 0*.*90* 1.00 (0.95–1.05)	N.A.
*Perceived barrier towards avoiding sun 12–15*	*p = 0*.*004* 1.09 (1.03–1.16)	*p = 0*.*014* 1.07 (1.01–1.14)
Ambient Sunhours/week	*p < 0*.*001* 1.03 (1.02–1.04)	*p < 0*.*001* 1.03 (1.02–1.04)
Age	*p = 0*.*001*	*p = 0*.*002*
15–24	2.8 (1.6–4.7)	2.9 (1.6–5.2)
25–34	2.1 (1.3–3.6)	2.2 (1.2–3.9)
35–44	1.9 (1.1–3.2)	1.5 (0.8–2.6)
45–54	1.4 (0.8–2.3)	1.2 (0.7–2.1)
55–65	Ref	Ref
Skin type	*p < 0*.*001*	*p < 0*.*001*
I	4.5 (2.4–8.5)	5.0 (2.5–10.1)
II	2.4 (1.6–3.5)	2.8 (1.8–4.4)
III / IV	Ref	Ref
Gender	*p = 0*.*020*	*p = 0*.*016*
Female	0.7 (0.5–0.9)	0.6 (0.4–0.9)
Male	Ref	Ref

Values are odds-ratios (OR) and 95% confidence intervals (CI). ^1^The model included gender, age groups, skin type, ambient number of sun hours /week, and scales of *knowledge deficit UV risk and melanoma* and *perceived barrier towards avoiding the sun between 12–15*.

## Discussion

We have identified new scales of knowledge deficit of areas related to UVR exposure, concept validated new and previous scales measuring knowledge, attitude and behavior related to UVR exposure and examined the scales association to objective measures of UVR exposure. Firstly, we have shown the correlation of a number of scales, predictors of protection behavior with our developed protection behavior scale. Secondly, we have shown that a *knowledge deficit of UVR risk* is directly associated to objectively measured UVR exposure and sunburn as well as is a barrier towards avoiding the sun between 12 and 15. Thirdly, we have identified a number of measures related to protection behavior and of those especially, but not surprisingly, the incorporation of routines in your protection behavior is an important predictor.

### Strength and limitations

The strengths of this study include a sample based on the Danish civil registration system, with very high participation and response rates and objective personal dosimetry measurements. The use of Rasch Scale validation ensures that scales are homogenous, free of differential item functioning and tested for local dependency. Contrary to traditional studies [41,42] of exposure to ultraviolet radiation based on questionnaires, this study reduced bias from recalling past sun exposure maximally by short measurement periods and short response periods. Limitations of the study are the wrist worn dosimeters which were previously shown to register about 50 percent of the ambient exposure (as received on top of the head) [43], however the bias introduced is assumed to be equally distributed and was described elsewhere [44]. Also lack of compliance with use of the dosimeters could introduce bias, however compliance was also previously described [44] and we did not register any directional bias. Persons wearing a dosimeter could be more aware of their behavior and this could change their behavior, however we previously tested this in a smaller intervention study and did not find an effect on wearing a dosimeter [45].

### Interpretation

The project has developed valid methods for measurements of the Danes sun-related behavior. A general monitor of the chosen parameters (knowledge, attitude and behavior) over time will increase our knowledge of the Danes sun-related behavior and be a tool for the SunSmart campaign to evaluate the campaign’s influence on decreasing the risk of skin cancer [46].

We have examined the associations between a number of scales covering potential important subjects for skin cancer prevention with the protection behavior scale. The protection behavior scale was further analyzed in a linear regression model where the incorporation of *protection behavior routine* revealed to be very important. *Perceived importance of sun protection and benefits of protection* were both significant in our model, as was also shown in the model proposed by *Branström*. We also examined *perceived severity of MM* and *Worry about developing MM*, *where the latter was included in the model by Branström*, *but did not find it significant*. *Attitude towards tan* inversely associated towards protection behavior in both our and Branström model. *Perceived barriers towards use of clothing* was included in our model where Branström used a combined barrier scale. In our model, however *perceived barrier towards avoiding sun between 12 and 15* was not included in the protection behavior model. *Perceived barrier towards avoiding sun between 12 and 15* was however inversely associated to exposure time.

To our knowledge, we are the first to report the associations of these scales and objective measures of UVR exposure. We are also the first to have developed knowledge deficit scales and showed that they are associated to the objective measures of the exposure. Other studies however have shown knowledge association to subjective measures of the exposure or precursors to the exposure [47,48]. Skin examination efficacy is not a behavior directly involved in the protection decision pathway, however it was associated to the exposure and may be linked to genetic disposition, own risk perception or likewise. Finally, we show scales directly associated to sunburn. We expected *Attitude towards tan* to be linked to sunburn as it was associated both to the protection behavior scale and to the exposure and because it was previously shown to be associated to the exposure [26,49]. The results might be a result of higher self-perceived sunburn threshold among this group or it could be a high aesthetic value to this group to tan but not to burn as they are both associated to exposure and protection. *Perceived barrier towards avoiding the sun between 12–15* and *Knowledge deficit of UV and risk of melanoma* were the only scales significantly associated to sunburn. Knowledge association towards sun related measures was previously shown to be ambiguous [26,47,50–52], while the *Perceived barrier towards avoiding the sun between 12–15* is in agreement with another finding we made in this data collection [30], that the exposure and sun avoidance may be much more important than the use of protection.

While our focus has primarily been to strengthen the tools for skin cancer prevention, we also examined knowledge about exposure to ultraviolet radiation and Vitamin D. Even though we did not find this knowledge associated with key indicators, our tool may also be useful to assess e.g. sufficient UVR exposure to reach sufficient levels of Vitamin D, an area where different opinions remain [5,53,54].

### Conclusion

This study is important for behavior in the sun as it provides items and scales associated to actual UVR exposure. The finding of possible efficiency of campaigning to give knowledge about risks associated to UVR exposure was suspected and now it is evident. The number 1 advice of the Danish Sun Safety Campaign is shade, which is also defined by avoiding the sun in the peak hours. These results emphasize the priority of the advice and to increase focus on this advice. Not being outdoor in the sun between 12 and 15 may be experienced as a barrier to many people. Shifting activities to occur outside the suns peak hours could be a possible approach that could be attacked by structural and campaign preventive measures.

## Materials and methods

In March 2013, a random sample of Danes in the age group 15–65 years was drawn from the Danish civil registration system. They were sent an invitation to participate in the study by mail in the end of April. To be eligible to the study they should be able to wear a personal dosimeter wristband for one week of their vacation in Denmark in the weeks 19–35 (May-August) and complete an electronic questionnaire afterwards. The invitees signed up on the project page http://www.mituv.dk (i.e. myuv.dk) and indicated available weeks. Participants who confirmed their participation by phone were sent a dosimeter including instructions and a prepaid envelope by ordinary mail. After participation they returned the dosimeter for data retrieval and were sent a questionnaire the following week.

The study population was chosen to be representative of the Danish population within gender, age groups (15–24, 25–34, 35–44, 45–54, 55–65) and region. The recruitment of the 15-17-year-olds required parental consent in which case the invitation letter was initially directed to one of the parents. Persons who have inquired not to be drawn for research projects were excluded from the sample. A more detailed description of the study population and size as well as data collection from personal dosimeters and questionnaires respectively was previously presented [30].

For questionnaire development and validation, a literature- and collective study, including questionnaires previously used for evaluations of interventions targeted at reducing UV exposure was conducted. On this background and on basis of ‘theory of planned behavior’ first version of the questionnaire was developed and tested by colleagues and professionals. In this process face validity (reasonable association of questions and objective) and contents validity (representativeness of questions in the area investigated) was evaluated. The second version of the questionnaire was tested on a cross-sectional sample of the population age 15–65 years [44]. Criteria validity for knowledge deficit and attitude were tested based on experienced presumptions according to the literature as well as accordance to behavioral measurements. Concept validity was tested by Rasch-analysis and reliability was tested by test-retest procedure. The questionnaire can be found in supplementary files. Sunburn reaction was self reported.

The ultraviolet dosimeters used for this study were electronic and originally developed at the University of Canterbury, New Zealand to digitally measure personal erythemal UV exposures in behavioral studies [55]. They are based on a visible-blind AlGaN photodiode and their spectral response and cosine response was previously described by Allen et al. (15). The version used here was re-designed and manufactured by Scienterra Ltd., New Zealand and used by Cargill et al., Wright et al. and Køster et al. [29,44,56]. The dosimeters were configured to make time stamped measurements at 30-second intervals from 7 am to 7 pm. Wristbands were attached to the dosimeters.

The dosimeters were calibrated against data from the Danish Meteorological Institute (Robertson Berger type instrument), and second degree polynomials were fitted for each badge, to convert logged data into erythemal effective units (UVI, SED). Danish Meteorological Institute also provided ambient UV data.

To examine correlation between attitude and knowledge deficit scales and registered time outdoors, we converted any 30-second UV measurement to 30 seconds of outdoor time. Then we summed measured time and dose for each participant and measurement week. Finally, number of days the dosimeter was worn was accounted for and average exposure per day was calculated. Attitude and knowledge deficit questions were primarily based on 5-point Likert scales. Increasing values of scales indicates a higher degree of the subject in question. For example, a higher scale score for use of protection indicates use of more protection. The complete questionnaire is found in supplementary materials S1 Appendix. The self-evaluated weather was determined with a single question on average cloud cover (1–5). Skin type was assigned according to Fitzpatrick[57] by self-evaluated skin tan/burn reaction upon season’s first exposure to the sun.

Descriptive statistics for continuous variables are presented as means. Differences between distribution of the variables was examined by t-test or anova as appropriate. Confidence intervals for the spearman correlation coefficients were calculated using Fisher’s transformation. Assumptions of linearity and homogeneity of variance were satisfied. The normal distribution of data was tested by QQplots and Shapiro-Wilks tests. Square root transformation of data was distributed normally, and used when data deviated from the normal distribution. Linear regression models were used to assess associations, where (objective measures of UVR exposure, protection behavior scale) were the independent variable respectively and to assess associations between the knowledge deficit and attitudinal scales respectively. Residuals were normally distributed. Logistic regression was used to assess associations between sunburn and examined scales. The scales were validated by testing unidimensionality, homogeneity, monotonocity, local independence, differential item functioning. General Log Linear Rasch Models (GLLRM) was applied. The project was sent to The National Committee on Health Research Ethics who decided that their approval was not necessary. Danish Data Protection Agency gave approval number 2012-41-0100. SAS 9.3 and Digram were used for the analyses.

## Supporting information

S1 Table Mean, item-scale and item-item correlation and item fit statistics for items included in final scales. (DOCX)

S1 Appendix Questionnaire applied in Danish, translated to English. Gross scales and applied value indicated. Only successfully validated scales applied in the final analysis.
(DOCX)
