# Supplementary material for: Knowledge deficit, attitude and behavior scales association to objective measures of sun exposure and sunburn in a Danish population based sample
Source: PLoS One. 2017 May 25;12(5):e0178190. doi: 10.1371/journal.pone.0178190 (PMC5444774; doi:10.1371/journal.pone.0178190)
Supplement: S1 Table — (DOCX) [file pone.0178190.s001.docx]

**Supplementary S1 table**

Mean, item-scale and item-item correlation and item fit statistics for items included in final scales.

| Item (item number)* | Mean 0-3 | Restscore corr. | Item-item corr-range | Outfit(SD) | p-value | Infit(SD) | p- value | Item-restscore gamma –observed/expected (SD) | p- value |
| --- | --- | --- | --- | --- | --- | --- | --- | --- | --- |
| **Sun Protection** |  |  |  |  |  |  |  |  |  |
| SPF15 (25) | 0.93 | 0.271 | 0.12-0.34 | 0.93 (0.07) | 0.31 | 0.99 (0.06) | 0.81 | 0.27 / 0.22 (0.05) | 0.30 |
| Longsleeves (27) | 0.97 | 0.495 | 0.17-0.83 | 1.00 (0.07) | 0.96 | 0.98 (0.06) | 0.71 | 0.50 / 0.49 (0.04) | 0.97 |
| Long pants (28) | 1.01 | 0.446 | 0.12-0.83 | 1.18 (0.11) | 0.12 | 1.09 (0.07) | 0.17 | 0.45 / 0.50 (0.04) | 0.19 |
| Cap (29) | 0.43 | 0.198 | 0.08-0.73 | 1.10 (0.13) | 0.43 | 0.99 (0.11) | 0.92 | 0.20 / 0.17 (0.06) | 0.63 |
| Full brimmed hat (30) | 0.12 | 0.371 | 0.14-0.73 | 1.07 (0.27) | 0.78 | 0.94 (0.21) | 0.77 | 0.37 / 0.31 (0.10) | 0.52 |
| Shade (31) | 1.18 | 0.331 | 0.08-0.54 | 1.01 (0.05) | 0.78 | 1.00 (0.06) | 0.94 | 0.33 / 0.34 (0.04) | 0.79 |
| Avoid sun between 12 & 15 (32) | 0.74 | 0.341 | 0.10-0.54 | 0.99 (0.07) | 0.87 | 1.00 (0.07) | 0.96 | 0.34 / 0.33 (0.04) | 0.84 |
| **Perceived importance of protection outdoor exposure** |  |  |  |  |  |  |  |  |  |
| Hat (50) | 1.95 | 0.58 | 0.45-0.54 | 1.08 (0.08) | 0.32 | 1.05 (0.07) | 0.49 | 0.58 / 0.58 (0.03) | 0.98 |
| Clothing (51) | 1.61 | 0.63 | 0.49-0.54 | 0.94 (0.08) | 0.41 | 0.95 (0.07) | 0.50 | 0.63 / 0.61 (0.03) | 0.52 |
| Sun avoidance (52) | 2.41 | 0.52 | 0.45-0.49 | 0.99 (0.07) | 0.95 | 0.99 (0.06) | 0.84 | 0.52 / 0.52 (0.03) | 0.84 |
| **Skin examination self-efficacy** |  |  |  |  |  |  |  |  |  |
| Self examination (76) | 1.20 | 0.83 | 0.59-0.87 | 0.55 (0.16) | 0.58 | 0.84 (0.22) | 0.47 | 0.83 / 0.78 (0.09) | 0.61 |
| Examination by family (77) | 1.61 | 0.80 | 0.48-0.87 | 0.57 (0.19) | 0.68 | 0.91 (0.25) | 0.71 | 0.80 / 0.76 (0.10) | 0.71 |
| Examination by professional (78) | 1.91 | 0.52 | 0.48-0.59 | 0.97 (0.30) | 0.27 | 1.26 (0.36) | 0.47 | 0.52 / 0.66 (0.14) | 0.31 |
| **Perceived efficiency of skin examination** |  |  |  |  |  |  |  |  |  |
| GP skin change (79) | 2.91 | 0.40 | 0.25-0.54 | 1.06 (0.06) | 0.29 | 1.08 (0.62) | 0.19 | 0.40 / 0.45 (0.36) | 0.21 |
| GP secure health (80) | 2.30 | 0.60 | 0.43-0.67 | 0.92 (0.06) | 0.20 | 0.90 (0.62) | 0.10 | 0.60 / 0.53 (0.33) | 0.03 |
| GP long life (81) | 2.09 | 0.56 | 0.38-0.67 | 0.99 (0.07) | 0.85 | 0.98 (0.65) | 0.74 | 0.56 / 0.54 (0.34) | 0.59 |
| Self skin change (82) | 3.02 | 0.53 | 0.40-0.57 | 0.98 (0.07) | 0.76 | 1.00 (0.60) | 0.95 | 0.53 / 0.53 (0.36) | 0.95 |
| Self control health (84) | 2.32 | 0.49 | 0.25-0.57 | 1.03 (0.06) | 0.66 | 1.02 (0.60) | 0.75 | 0.49 / 0.47 (0.35) | 0.70 |
| **Perceived benefits of protection behavior** |  |  |  |  |  |  |  |  |  |
| Sunscreen prevents cancer (85) | 3.08 | 0.68 | 0.46-0.65 | 0.88 (0.07) | 0.09 | 0.87 (0.07) | 0.06 | 0.68 / 0.62 (0.03) | 0.06 |
| Sunscreen look young (86) | 2.58 | 0.67 | 0.40-0.72 | 1.04 (0.07) | 0.59 | 1.02 (0.07) | 0.73 | 0.67 / 0.66 (0.03) | 0.71 |
| SPF15 prevents burn (87) | 3.07 | 0.60 | 0.37-0.65 | 1.16 (0.07) | 0.02 | 1.07 (0.07) | 0.31 | 0.60 / 0.61 (0.03) | 0.68 |
| Clothes look young (90) | 2.55 | 0.74 | 0.49-0.74 | 0.95 (0.08) | 0.50 | 0.98 (0.07) | 0.74 | 0.74 / 0.74 (0.02) | 0.88 |
| Clothes prevents cancer (88) | 3.08 | 0.69 | 0.40-0.63 | 0.94 (0.08) | 0.46 | 1.00 (0.07) | 0.99 | 0.69 / 0.67 (0.03) | 0.56 |
| Hat prevents burn (89) | 2.99 | 0.57 | 0.40-0.63 | 1.07 (0.07) | 0.35 | 1.04 (0.07) | 0.59 | 0.57 / 0.61 (0.03) | 0.24 |
| Avoid sun 12-15 prevents skinageing (91) | 2.63 | 0.67 | 0.42-0.74 | 1.08 (0.07) | 0.25 | 1.06 (0.07) | 0.39 | 0.67 / 0.68 (0.03) | 0.73 |
| Use of shade prevents skin cancer (93) | 3.02 | 0.66 | 0.37-0.62 | 0.95 (0.06) | 0.45 | 0.95 (0.06) | 0.41 | 0.66 / 0.61 (0.03) | 0.11 |
| **Perceived barriers of Skin Examination** |  |  |  |  |  |  |  |  |  |
| GP expensive timeconsuming (96) | 1.71 | 0.28 | 0.12-0.43 | 1.12 (0.05) | 0.02 | 1.10 (0.05) | 0.06 | 0.28 / 0.33 (0.04) | 0.19 |
| GP_uncomfortable (97) | 1.06 | 0.54 | 0.23-0.62 | 0.95 (0.16) | 0.73 | 0.93 (0.06) | 0.29 | 0.54 / 0.52 (0.03) | 0.59 |
| GP Worry (98) | 1.45 | 0.50 | 0.20-0.62 | 1.03 (0.08) | 0.69 | 1.08 (0.06) | 0.23 | 0.51 / 0.54 (0.03) | 0.33 |
| Self examination Worry (100) | 1.54 | 0.42 | 0.12-0.58 | 1.03 (0.07) | 0.68 | 1.03 (0.07) | 0.64 | 0.42 / 0.43 (0.04) | 0.90 |
| Not good at self examination (101) | 2.39 | 0.28 | 0.22-0.26 | 0.90 (0.05) | 0.04 | 0.91 (0.05) | 0.06 | 0.28 / 0.20 (0.04) | 0.02 |
| **Perceived barriers sunscreen** |  |  |  |  |  |  |  |  |  |
| Difficult  (102) | 1.072 | 0.56 | 0.34-0.73 | 1.15 (0.07) | 0.04 | 1.03 (0.06) | 0.62 | 0.56 / 0.57 (0.03) | 0.75 |
| Expensive (104) | 1.559 | 0.37 | 0.34-0.39 | 1.04 (0.06) | 0.47 | 1.00 (0.05) | 0.96 | 0.37 / 0.35 (0.04) | 0.61 |
| Disturbing (106) | 1.081 | 0.60 | 0.39-0.73 | 0.97 (0.07) | 0.63 | 0.96 (0.07) | 0.54 | 0.60 / 0.58 (0.03) | 0.60 |
| **Perceived barriers clothing** |  |  |  |  |  |  |  |  |  |
| Difficult (109) | 1.861 | 0.58 | 0.46-0.59 | 1.02 (0.07) | 0.75 | 0.99 (0.06) | 0.91 | 0.58 / 0.54 (0.03) | 0.26 |
| Inconvenient (110) | 2.395 | 0.56 | 0.37-0.59 | 0.96 (0.07) | 0.55 | 0.95 (0.06) | 0.45 | 0.56 / 0.54 (0.03) | 0.64 |
| Embarrasing(111) | 1.360 | 0.54 | 0.37-0.65 | 1.05 (0.07) | 0.49 | 1.05 (0.06) | 0.45 | 0.54 / 0.57 (0.03) | 0.33 |
| Uncomfortable (113) | 1.613 | 0.64 | 0.51-0.65 | 0.96 (0.08) | 0.57 | 0.93 (0.07) | 0.30 | 0.64 / 0.59 (0.03) | 0.10 |
| Disturbing(114) | 1.661 | 0.53 | 0.44-0.54 | 1.07 (0.07) | 0.35 | 1.03 (0.07) | 0.68 | 0.53 / 0.53 (0.03) | 0.98 |
| **Perceived Barriers sun avoidance** |  |  |  |  |  |  |  |  |  |
| Difficult (125) | 2.102 | 0.70 | 0.48-0.76 | 1.16 (0.13) | 0.21 | 1.01 (0.09) | 0.90 | 0.70 / 0.70 (0.03) | 0.91 |
| Inconvenient(126) | 2.148 | 0.77 | 0.54-0.76 | 1.02 (0.11) | 0.86 | 0.87 (0.09) | 0.13 | 0.77 / 0.72 (0.03) | 0.04 |
| Suit well(127) | 2.351 | 0.49 | 0.25-0.54 | 0.94 (0.07) | 0.41 | 0.98 (0.07) | 0.74 | 0.49 / 0.47 (0.04) | 0.50 |
| Disturbing (128) | 1.979 | 0.48 | 0.25-0.60 | 1.05 (0.07) | 0.52 | 1.05 (0.07) | 0.47 | 0.48 / 0.51 (0.03) | 0.38 |
| **Perceived barriers hat** |  |  |  |  |  |  |  |  |  |
| Embarrasing (119) | 1.593 | 0.75 | 0.59-0.80 | 1.08 (0.07) | 0.31 | 1.05 (0.07) | 0.49 | 0.75 / 0.73 (0.02) | 0.39 |
| Uncomfortable (118) | 1.855 | 0.76 | 0.60-0.80 | 0.95 (0.08) | 0.50 | 0.97 (0.07) | 0.70 | 0.76 / 0.73 (0.02) | 0.30 |
| Disturbing (122) | 1.780 | 0.61 | 0.59-0.60 | 0.99 (0.08) | 0.90 | 0.99 (0.07) | 0.93 | 0.61 / 0.56 (0.03) | 0.30 |
| **Severity of melanoma** |  |  |  |  |  |  |  |  |  |
| Easy to cure (131) | 1.252 | 0.41 | 0.44-0.45 | 1.03 (0.08) | 0.70 | 0.97 (0.07) | 0.65 | 0.41 / 0.39 (0.04) | 0.63 |
| Serious (132) | 0.655 | 0.72 | 0.45-0.84 | 0.89 (0.17) | 0.51 | 0.96 (0.10) | 0.69 | 0.72 / 0.68 (0.03) | 0.31 |
| A risk to me (133) | 0.910 | 0.68 | 0.44-0.84 | 1.14 (0.09) | 0.12 | 1.06 (0.08) | 0.51 | 0.68 / 0.70 (0.03) | 0.48 |
| **General risk perception** |  |  |  |  |  |  |  |  |  |
| Risk tobacco (134) | 0.301 | 0.64 | 0.32-0.78 | 0.80 (0.20) | 0.20 | 0.91 (0.10) | 0.34 | 0.64 / 0.58 (0.04) | 0.14 |
| Risk weight (135) | 0.619 | 0.65 | 0.38-0.79 | 1.17 (0.11) | 0.10 | 1.10 (0.07) | 0.14 | 0.65 / 0.68 (0.03) | 0.40 |
| Risk blodPressure (136) | 0.747 | 0.67 | 0.44-0.79 | 0.87 (0.08) | 0.10 | 0.94 (0.07) | 0.37 | 0.67 / 0.63 (0.03) | 0.27 |
| Risk low fruit (137) | 1.759 | 0.45 | 0.32-0.49 | 1.05 (0.06) | 0.50 | 1.02 (0.06) | 0.76 | 0.45 / 0.46 (0.04) | 0.77 |
| Risk UV exposure(138) | 0.994 | 0.43 | 0.38-0.49 | 1.02 (0.06) | 0.70 | 1.02 (0.06) | 0.75 | 0.43 / 0.44 (0.04) | 0.90 |
| **Worry about Melanoma** |  |  |  |  |  |  |  |  |  |
| Worry likelyhood me (140) | 1.660 | 0.62 | 0.42-0.71 | 1.08 (0.08) | 0.31 | 1.02 (0.07) | 0.82 | 0.62 / 0.63 (0.03) | 0.85 |
| Worry famous (141) | 1.279 | 0.69 | 0.52-0.71 | 1.06 (0.10) | 0.53 | 0.98 (0.08) | 0.82 | 0.69 / 0.68 (0.03) | 0.74 |
| Cancer horrible (142) | 0.592 | 0.44 | 0.42-0.52 | 0.98 (0.10) | 0.86 | 1.02 (0.07) | 0.79 | 0.44 / 0.45 (0.04) | 0.89 |
| **Attitude tanning – own** |  |  |  |  |  |  |  |  |  |
| Color is healthy (147) | 2.00 | .36 | .20-.63 | 1.09 (0.05) | .09 | 1.09 (0.05) | .10 | .36 / .41 (.04) | .13 |
| Tan makes Me look healthy (148) | 2.56 | .59 | .28-.75 | 1.20 (0.09) | **.02** | 1.11 (0.05) | .08 | .59 / .62 (.03) | .29 |
| Tan makes me more comfortable (152) | 2.60 | .71 | .39-.87 | 0.91 (0.08) | .27 | 0.86 (0.05) | **.04** | .71 / .65 (.03) | **.02** |
| I like Color (160) | 2.95 | .79 | .41-.87 | 0.78 (0.09) | **.02** | 0.84 (0.05) | **.04** | .79 / .71 (.03) | **.01** |
| I do not care about tan (162) | 2.08 | .56 | .26-.67 | 0.90 (0.06) | .06 | 0.86 (0.05) | **.01** | .56 / .45 (.03) | **.00** |
| I do not like to lay in the sun (163) | 2.01 | .38 | .20-.52 | 1.17 (0.06) | .00 | 1.15 (0.05) | **.01** | .38 / .44 (.03) | .05 |
| I do not like to be pale (164) | 2.79 | .59 | .30-.78 | 1.07 (0.08) | .37 | 1.02 (0.05) | .84 | .59 / .53 (.04) | .08 |
| **Knowledge on uv behavior** |  |  |  |  |  |  |  |  |  |
| **Knowledge about UV penetration** |  |  |  |  |  |  |  |  |  |
| UV_shade (169) | 1.553 | 0.53 | 0.38-0.65 | 1.23 (0.10) | 0.02 | 1.06 (0.07) | 0.40 | 0.53 / 0.56 (0.04) | 0.41 |
| UV sunbathe (170) | 0.498 | 0.64 | 0.41-0.90 | 0.91 (0.11) | 0.41 | 1.00 (0.07) | 0.96 | 0.64 / 0.63 (0.04) | 0.72 |
| UV_water (171) | 0.623 | 0.61 | 0.38-0.90 | 1.03 (0.11) | 0.77 | 0.99 (0.08) | 0.85 | 0.61 / 0.61 (0.03) | 0.89 |
| UV_cloud (172) | 1.316 | 0.77 | 0.62-0.76 | 0.99 (0.09) | 0.88 | 0.93 (0.08) | 0.33 | 0.77 / 0.71 (0.03) | 0.06 |
| UV_rain(173) | 1.783 | 0.58 | 0.41-0.76 | 1.19 (0.10) | 0.05 | 0.99 (0.06) | 0.81 | 0.58 / 0.59 (0.03) | 0.70 |
| **Knowledge about UV types** |  |  |  |  |  |  |  |  |  |
| UVA cancer (174) | 1.364 | 0.79 | 0.55-0.87 | 1.12 (0.20) | 0.54 | 0.90 (0.16) | 0.53 | 0.79 / 0.76 (0.03) | 0.38 |
| UVB cancer (175) | 1.487 | 0.89 | 0.66-0.87 | 0.79 (0.22) | 0.34 | 0.89 (0.11) | 0.31 | 0.89 / 0.85 (0.03) | 0.17 |
| UVC cancer (176) | 1.662 | 0.61 | 0.55-0.66 | 0.70 (0.22) | 0.17 | 0.90 (0.14) | 0.47 | 0.61 / 0.58 (0.05) | 0.51 |
| **Knowledge about UV and d-vitamin** |  |  |  |  |  |  |  |  |  |
| Vit D shade (185) | 1.693 | 0.30 | 0.14-0.41 | 1.06 (0.05) | 0.29 | 1.08 (0.05) | 0.14 | 0.30 / 0.35 (0.04) | 0.14 |
| Vit D 1215 (186) | 1.030 | 0.62 | 0.27-0.79 | 0.93 (0.08) | 0.37 | 0.95 (0.07) | 0.47 | 0.62 / 0.58 (0.03) | 0.15 |
| Vit D (187) sunbathe | 1.104 | 0.68 | 0.39-0.79 | 0.86 (0.23) | 0.52 | 0.92 (0.07) | 0.20 | 0.68 / 0.63 (0.03) | 0.06 |
| Vit D (188) sunscreen | 1.181 | 0.54 | 0.29-0.62 | 0.95 (0.07) | 0.49 | 0.94 (0.06) | 0.34 | 0.54 / 0.48 (0.04) | 0.13 |
| Vit D 20 minuts(189) | 1.471 | 0.49 | 0.17-0.81 | 1.00 (0.06) | 1.00 | 1.00 (0.06) | 0.99 | 0.49 / 0.49 (0.04) | 0.94 |
| Vit D 3hours (190) | 1.594 | 0.48 | 0.14-0.81 | 1.01 (0.06) | 0.84 | 1.01 (0.06) | 0.84 | 0.48 / 0.49 (0.03) | 0.95 |
| **Knowledge about melanoma and UVR risk** |  |  |  |  |  |  |  |  |  |
| Indoor tanning (177) | 0.41 | 0.59 | 0.53-0.67 | 1.06 (0.09) | 0.48 | 1.08 (0.07) | 0.20 | 0.59 / 0.60 (0.04) | 0.93 |
| Travel to sunny (178) destination | 0.83 | 0.61 | 0.52-0.65 | 1.00 (0.06) | 0.97 | 1.03 (0.06) | 0.57 | 0.61 / 0.63 (0.03) | 0.53 |
| Sunburn in adulthood (180) | 0.91 | 0.63 | 0.52-0.71 | 0.98 (0.05) | 0.71 | 0.98 (0.05) | 0.67 | 0.63 / 0.61 (0.03) | 0.50 |
| Outdoor unprotected (181) | 0.57 | 0.73 | 0.58-0.85 | 1.02 (0.09) | 0.84 | 0.98 (0.07) | 0.81 | 0.73 / 0.72 (0.03) | 0.92 |
| Sunbathing without sunscreen (183) | 0.52 | 0.77 | 0.60-0.85 | 0.87 (0.11) | 0.21 | 0.91 (0.07) | 0.20 | 0.77 / 0.73 (0.03) | 0.18 |
| Outdoor work (184) | 1.16 | 0.63 | 0.53-0.65 | 1.00 (0.06) | 0.97 | 1.01 (0.06) | 0.92 | 0.63 / 0.64 (0.03) | 0.63 |
| **Routine** |  |  |  |  |  |  |  |  |  |
| Routine sunscreen (107) | 2.09 | .15 | .09-.17 | 1.01 (0.05) | 0.85 | 1.00 (0.05) | 1.00 | 0.15 / 0.14 (0.04) | .69 |
| Routine Hat (123) | 2.65 | .31 | .09-.40 | 1.03 (0.06) | 0.65 | 1.00 (0.06) | 0.99 | 0.31 / 0.28 (0.04) | .42 |
| Routine outdoor 12 &15 (129) | 2.45 | .34 | .14-.43 | 1.03 (0.06) | 0.63 | 1.02 (0.06) | 0.79 | 0.34 / 0.33 (0.04) | .75 |
| Routine clothing (115) | 2.41 | .45 | .17-.43 | 1.05 (0.06) | 0.38 | 0.99 (0.06) | 0.82 | 0.45 / 0.41 (0.04) | .26 |
| **Perceived barrier not Tanning** |  |  |  |  |  |  |  |  |  |
| Tan unachievementsunscreen (108) | 1.35 | .55 | .50-.53 | 1.11 (0.06) | 0.05 | 1.10 (0.06) | 0.08 | 0.55 / 0.57 (0.03) | .60 |
| Tan unachievement Hat (124) | 1.42 | .60 | .53-.61 | 1.00 (0.07) | 0.96 | 1.01 (0.07) | 0.87 | 0.60 / 0.59 (0.03) | .66 |
| Tan unachievementoutdoor 12 &15 (130) | 1.61 | .66 | .52-.63 | 0.99 (0.07) | 0.82 | 0.97 (0.07) | 0.59 | 0.66 / 0.62 (0.03) | .11 |
| Tan unachievement clothing (116) | 1.96 | .61 | .50-.63 | 0.92 (0.06) | 0.15 | 0.94 (0.06) | 0.24 | 0.61 / 0.56 (0.03) | .10 |

***Corresponding item numbers are indicated in the questionnaire**
