# Supplementary material for: Knowledge deficit, attitude and behavior scales association to objective measures of sun exposure and sunburn in a Danish population based sample
Source: PLoS One. 2017 May 25;12(5):e0178190. doi: 10.1371/journal.pone.0178190 (PMC5444774; doi:10.1371/journal.pone.0178190)
Supplement: S1 Appendix — Gross scales and applied value indicated. Only successfully validated scales applied in the final analysis. (DOCX) [file pone.0178190.s002.docx]

| **Dear participant,**  **Thank you for answering our questionnaire. Your help is important for our work of finding out how to avoid skin cancer.**  **There is no right or wrong answers. However, it is very important for us that you answer all the questions. It will take about 20-30 minutes to fulfill the questionnaire. In the questions, you can see how far you are.**  **The questions are about behavior and attitudes in and about the sun when you are in Denmark. It can be hard to remember exactly what you did, but we ask you for your best estimate.**  **If you are prevented from continuing during the questionnaire, your answers will be saved. You can proceed if you save the link in the e-mail. You just have to click along to the question you stopped at.**   **Thank you!** |
| --- |

What type of media do you use to answer this questionnaire? [1]

(1) ❑ PC

(2) ❑ Tablet, e.g. iPad

(3) ❑ Smartphone

(4) ❑ Other __________

What is your gender? [2]

(1) ❑ Female

(2) ❑ Male

When were you born? [3]

____ (birthyear)

In what region do you live? [4]

(1) ❑ The Capital Region of Denmark

(2) ❑ Region Zealand

(3) ❑ The North Denmark Region

(4) ❑ Central Denmark Region

(5) ❑ Region of Southern Denmark

(6) ❑ I live in another country

(7) ❑ Don’t know

What is your highest terminated education? [5]

(1) ❑ Primary education (e.g. public school)

(2) ❑ Vocational college (e.g. EUD)

(3) ❑ High school (e.g. gymnasium, HF, HTX, HHX)

(6) ❑ Higher education (up to 2 years)

(7) ❑ Higher education (2 to 4½ years)

(8) ❑ Higher education (5 years or more)

(9) ❑ Other __________

Do you have children under the age of 18 living at home? [6]

(1) ❑ Yes

(2) ❑ No

What is your marital status? [7]

(1) ❑ Married

(2) ❑ Living together with a partner

(3) ❑ In a relationship but not cohabiting

(4) ❑ Single

(5) ❑ Other

Scale 1 – Risk Group

In the beginning of the sun season, when your tan hasn’t changed yet. How does your skin react to the first sun exposure? [8]

Value

(1) ❑ I always get a sunburn 2

(2) ❑ I sometimes get a sunburn 1

(3) ❑ I seldom get a sunburn 0

(4) ❑ I never get a sunburn 0

In the beginning of the sun season, when your tan hasn’t changed yet. How does your skin react to the first sun exposure? [9] Value

(1) ❑ I never get a tan 2

(2) ❑ I get slightly tanned 1

(3) ❑ I get tanned 0

(4) ❑ I get very tanned 0

What color are your eyes? [10] Value

(1) ❑ Blue 2

(2) ❑ Grey 1

(3) ❑ Green 1

(4) ❑ Brown 0

What was your natural hair color when you were 18 years old? [11]

Value

(1) ❑ Red 2

(2) ❑ Blond 2

(3) ❑ Light brown 1

(4) ❑ Dark brown 0

(5) ❑ Black 0

Do you have freckles? [12] Value

(1) ❑ Yes, many 2

(2) ❑ Yes, few 1

(3) ❑ No 0

How many moles (any pigmentation of the skin that is different from the surrounding area) do you have, that are larger than 6mm in diameter (equivalent to the tip of a pencil)? [13]

Value

(1) ❑ None 0

(2) ❑ 1-2 1

(3) ❑ 3-5 1

(4) ❑ 6-10 2

(5) ❑ 11 or more 2

Have you or any of the members of your nearest family ever been diagnosed with skin cancer (basal-cell, squamous-cell and melanoma)? [14] Value

(1) ❑ No, none in my family 0

(2) ❑ Yes, 1 family member 1

(3) ❑ Yes, several family members 2

| Here follows some questions about the week, in which you participated in the uv-measurement. Think back to the week you wore the uv-device. |
| --- |

How many hours were you outside in average pr. day in the measure-week?

|  | I was not outside in this period | 0-1 hour | 1-2 hours | 2-3 hours | 3-4 hours |
| --- | --- | --- | --- | --- | --- |
| 7 to 11 am [15] | (1) ❑ | (2) ❑ | (3) ❑ | (4) ❑ | (5) ❑ |
| 11 am to 3 pm [16] | (1) ❑ | (2) ❑ | (3) ❑ | (4) ❑ | (5) ❑ |
| 3 pm to 7 pm [17] | (1) ❑ | (2) ❑ | (3) ❑ | (4) ❑ | (5) ❑ |

Value 0 0.5 1.5 2.5 3.5

Value 0 1 2 3 4

How many hours were you outdoors in average pr. day in the period from 7 am to 7 pm in the measure-week? [18]

__

Scale 2 – Burn Frequency

In the measure-week, to which degree did you get a sunburn in the following places? (a sunburn is any form of redness, uncomfortableness, pain or blister on the skin lasting longer than 12 hours after staying in the sun)

|  | Not sunburned | A little sunburned | Some extent of sunburn | Sunburned a lot | Very much sunburned |
| --- | --- | --- | --- | --- | --- |
| Face, neck, hands, or feet[19] | (1) ❑ | (2) ❑ | (3) ❑ | (4) ❑ | (5) ❑ |
| Arms[20] | (1) ❑ | (2) ❑ | (3) ❑ | (4) ❑ | (5) ❑ |
| Legs[21] | (1) ❑ | (2) ❑ | (3) ❑ | (4) ❑ | (5) ❑ |
| Back[22] | (1) ❑ | (2) ❑ | (3) ❑ | (4) ❑ | (5) ❑ |
| Stomach [23] | (1) ❑ | (2) ❑ | (3) ❑ | (4) ❑ | (5) ❑ |
| Shoulders [24] | (1) ❑ | (2) ❑ | (3) ❑ | (4) ❑ | (5) ❑ |

Value 0 1 2 2 2
Sunburn in any bodypart was regarded as sunburned and dichotomized.

In the measure-week, for how much of the time did you use the following methods of sun protection outside in sunny weather between 12 and 3 pm?

Skala3 Protection Scale

|  | Never - 0% of the time | Seldom- 10-20% of the time | Sometimes - 30-50% of the time | Often - 60-80% of the time | Always - 90-100% of the time |
| --- | --- | --- | --- | --- | --- |
| Used sunscreen with high protection – SPF 15 or higher [25] | (1) ❑ | (2) ❑ | (3) ❑ | (5) ❑ | (6) ❑ |
| Used sunscreen with medium or low protection – lower than SPF 15 [26] | (1) ❑ | (2) ❑ | (3) ❑ | (5) ❑ | (6) ❑ |
| Wore clothes that protected against the sun – longsleeved blouses or similar [27] | (1) ❑ | (2) ❑ | (3) ❑ | (5) ❑ | (6) ❑ |
| Wore clothes that protected against the sun – long trousers or similar [28] | (1) ❑ | (2) ❑ | (3) ❑ | (5) ❑ | (6) ❑ |
| Wore a cap or other headwear that only has a shadow in front [29] | (1) ❑ | (2) ❑ | (3) ❑ | (5) ❑ | (6) ❑ |
| Wore a wide brimmed hat that has a shadow all the way around [30] | (1) ❑ | (2) ❑ | (3) ❑ | (5) ❑ | (6) ❑ |
| Stayed in the shades [31] | (1) ❑ | (2) ❑ | (3) ❑ | (5) ❑ | (6) ❑ |
| Tried to be indoors between 12 and 3 pm [32] | (1) ❑ | (2) ❑ | (3) ❑ | (5) ❑ | (6) ❑ |

Value 0 1 2 3 3

Skala 4 - Outdoor Sun

How many times did you sunbathe (i.e. stay in the sun, partially dressed, to get a tan) in Denmark in the measure-week? [33] Value

(1) ❑ Never 0

(2) ❑ 1 time 1

(3) ❑ 2-3 times 2

(4) ❑ 4-5 times 3

(5) ❑ 6 times or more 4

How many times did you sit or lay in the sun in the measure-week? [34]

Value

(1) ❑ Never 0

(2) ❑ 1 time 1

(3) ❑ 2-3 times 2

(4) ❑ 4-5 times 3

(6) ❑ 6 or more times 4

How was the weather in the measure-week? [35]

(1) ❑ Sunshine most of the time

(2) ❑ Sunshine and some clouds

(3) ❑ Equal amounts of sunshine and clouds

(4) ❑ Cloudy and some sunshine

(5) ❑ Cloudy most of the time (some rain)

(6) ❑ Don’t remember

| In the measure-week, you received a UV-measurement device from the Danish Cancer Society. Here follows some questions about the use of the device. |
| --- |

How many days did you wear UV-measurement device? [36]

(1) ❑ 0

(2) ❑ 1

(3) ❑ 2

(4) ❑ 3

(5) ❑ 4

(6) ❑ 5

(7) ❑ 6

(8) ❑ 7

Out of those days, you wore the device, how much of the time between 7 am and 7 pm did you wear the device when you were outside? [37]

(1) ❑ 1/4 of the time

(2) ❑ Half of the time

(3) ❑ 3/4 of the time

(4) ❑ All the time

When did you wear the UV-measurement device? [38]

(1) ❑ I put it on in the morning and wore it all day (except in the shower or while swimming), and took it off again in the evening

(2) ❑ I wore it most of the time but took it off at special occasions

(3) ❑ I only wore it when I went out

(4) ❑ I only wore it if the sun was shining

When you wore the device, did you pay attention to it? [39]

(1) ❑ Yes, I was aware that it should be free from sleeves etc., but I didn’t change my behavior because of it

(2) ❑ Yes, I gave more thought to whether I was in the sun or not than I would have done without the device on my arm

(3) ❑ No, I didn’t pay attention to it at all

While you wore the UV-measuement device, did people around you notice it and ask you what it was for? [40]

(1) ❑ Yes, many

(2) ❑ Yes, some

(3) ❑ No, no one

Please note how many days in the measure-week, you did the following:

(The summed amount should be 7)

| I was off from work/on vacation | __________[41] |
| --- | --- |
| I was indoors in my workplace | __________[42] |
| I was outdoors in my workplace | __________[43] |

Skala 5 - Attitude outdoor protection

Please note how much you disagree or agree on the following – When the sun is shining …

|  | Disagree a lot | Disagree | Neither disagree nor agree | Agree | Agree a lot |
| --- | --- | --- | --- | --- | --- |
| I use every possibily to be outdoors [44] | (1) ❑ | (2) ❑ | (3) ❑ | (4) ❑ | (5) ❑ |
| I don’t think about sunprotecting myself [45] | (1) ❑ | (2) ❑ | (3) ❑ | (4) ❑ | (5) ❑ |
| I find it hard not to be outdoors between 12 and 3 pm [46] | (1) ❑ | (2) ❑ | (3) ❑ | (4) ❑ | (5) ❑ |

Value 0 1 2 3 4

Skala 6 – Risk Sun

Within the last 12 months, how many weeks were you on vacation in a place where the sun usually shines most of the time? [47] Value

(1) ❑ Never 0

(2) ❑ Less than a week 1

(3) ❑ 1 week 2

(4) ❑ 2-3 weeks 3

(6) ❑ 4 weeks or more 4

How often did you use a sunbed within the last 12 months? [48] Value

(1) ❑ Several times a week 4

(2) ❑ 1 time a week 4

(3) ❑ 2-3 times a week 3

(4) ❑ 1-11 times within the last year 2

(5) ❑ I did not use a sunbed within the last 12 months but

I have previously used sunbeds

1

(6) ❑ I have never used a sunbed 0

Do you work (or worked if you are retired) mostly indoors or outdoors? [49] Value

(1) ❑ Only indoors 0

(2) ❑ Mostly indoors 1

(3) ❑ Equal amounts of indoor and outdoor 2

(4) ❑ Mostly outdoors 3

(5) ❑ Only outdoors 4

| Please note to which degree you diagree or agree |
| --- |

Skala 7 – Attitude importance of protection

If you are on vacation in a southern destination, e.g. in Spain, it is important to …

|  | Diasgree a lot | Disagree | Neither disagree or agree | Agree | Agree a lot |
| --- | --- | --- | --- | --- | --- |
| Use a hat [50] | (1) ❑ | (2) ❑ | (3) ❑ | (4) ❑ | (5) ❑ |
| Wear clothes that cover arms and legs [51] | (1) ❑ | (2) ❑ | (3) ❑ | (4) ❑ | (5) ❑ |
| Avoid the sun between 12 and 3 pm [52] | (1) ❑ | (2) ❑ | (3) ❑ | (4) ❑ | (5) ❑ |
| Use sunscreen [53] | (1) ❑ | (2) ❑ | (3) ❑ | (4) ❑ | (5) ❑ |

If you are on vacation in Denmark it is important to …

|  | Diasgree a lot | Disagree | Neither disagree or agree | Agree | Agree a lot |
| --- | --- | --- | --- | --- | --- |
| Use a hat [54] | (1) ❑ | (2) ❑ | (3) ❑ | (4) ❑ | (5) ❑ |
| Wear clothes that cover arms and legs [55] | (1) ❑ | (2) ❑ | (3) ❑ | (4) ❑ | (5) ❑ |
| Avoid the sun between 12 and 3 pm [56] | (1) ❑ | (2) ❑ | (3) ❑ | (4) ❑ | (5) ❑ |
| Use sunscreen [57] | (1) ❑ | (2) ❑ | (3) ❑ | (4) ❑ | (5) ❑ |

Value 0 1 2 3 4

| Here follows some questions about sunscreen. If you never use sunscreen, you will automatically move on to the next category of questions. |
| --- |

Do you use sunscreen? [58]

(1) ❑ Yes

(2) ❑ No, never

Skala 8 – Use of Sunscreen

When you use sunscreen, when do you normally put it on? [59] Value

(1) ❑ Before I go outside (before I leave my home), or in the car ("going somewhere”) 3

(2) ❑ As soon as I am in the sun (as soon as I reach my destination – e.g. by the pool or in the park) 2

(3) ❑ After being in the sun for a while, but before I realize that my skin has turned red 1

(4) ❑ If I realize that my skin has turned red 0

When you use sunscreem, how much do you normally apply? [60] Value

(1) ❑ I use a very small amount – a very thin layer 0

(2) ❑ I use a little – a thin layer 1

(3) ❑ I use a moderate layer – enough to cover everything 2

(4) ❑ I use a lot – a thick layer 3

How often would you say, you re-apply your sunscreen? [61] Value

(1) ❑ About once an hour 3

(2) ❑ Every 2-3 hours 2

(3) ❑ Rarer than every 3 hours 1

(4) ❑ I only apply once a day 0

When you use sunscreen in Denmark, what SPF do you use? [62] Value

(1) ❑ Lower than SPF 15 0

(2) ❑ SPF 15-20 1

(3) ❑ SPF 21-29 2

(4) ❑ SPF 30 or higher 3

When you use sunscreen on vacations to sunny destinations, what SPF do you use? [63] Value

(1) ❑ Lower than SPF 15 0

(2) ❑ SPF 15-20 1

(3) ❑ SPF 21-29 2

(4) ❑ SPF 30 or higher 3

Scale 24 – Sunscreen bodypart

To which degree do you use sunscreen in the following places?

|  | Not at all | To a small degree | To some degree | To a high degree | To a very high degree |
| --- | --- | --- | --- | --- | --- |
| Face, neck, hands, or feet [64] | (1) ❑ | (2) ❑ | (3) ❑ | (4) ❑ | (5) ❑ |
| Arms [65] | (1) ❑ | (2) ❑ | (3) ❑ | (4) ❑ | (5) ❑ |
| Legs [66] | (1) ❑ | (2) ❑ | (3) ❑ | (4) ❑ | (5) ❑ |
| Back [67] | (1) ❑ | (2) ❑ | (3) ❑ | (4) ❑ | (5) ❑ |
| Stomach [68] | (1) ❑ | (2) ❑ | (3) ❑ | (4) ❑ | (5) ❑ |
| Shoulders [69] | (1) ❑ | (2) ❑ | (3) ❑ | (4) ❑ | (5) ❑ |

Value 0 1 2 3 3

Which protection do you use in the following parts of the body?

|  | Only sunscreen | Only clothes/hat | Both sunscreen and clothes/hat | Neither |
| --- | --- | --- | --- | --- |
| Face, neck, hands, or feet[70] | (1) ❑ | (2) ❑ | (3) ❑ | (4) ❑ |
| Arms[71] | (1) ❑ | (2) ❑ | (3) ❑ | (4) ❑ |
| Legs[72] | (1) ❑ | (2) ❑ | (3) ❑ | (4) ❑ |
| Back[73] | (1) ❑ | (2) ❑ | (3) ❑ | (4) ❑ |
| Stomach[74] | (1) ❑ | (2) ❑ | (3) ❑ | (4) ❑ |
| Shoulders[75] | (1) ❑ | (2) ❑ | (3) ❑ | (4) ❑ |

Scale 9 – skin examination self efficacy

| **Here follows some questions about skin examinations** |
| --- |

|  | Weekly | Monthly | Every half year | Yearly or rarer | Never |
| --- | --- | --- | --- | --- | --- |
| How often do you examine yourself thoroughly for changes and signs of skin cancer? [76] | (2) ❑ | (3) ❑ | (4) ❑ | (5) ❑ | (7) ❑ |
| How often do you ask a family member (or a friend) to examine your skin thoroughly for changes and signs of skin cancer? [77] | (2) ❑ | (3) ❑ | (4) ❑ | (5) ❑ | (7) ❑ |
| How often do you go to a professional (general practitioner or dermatologist) to get a full skin examination? [78] | (2) ❑ | (3) ❑ | (4) ❑ | (5) ❑ | (7) ❑ |

Value 0 1 2 3 4

Scale 10 – perceived efficiency skin examination

To which degree do you disagree or agree on the following:

|  | Diasgree a lot | Disagree | Neither disagree or agree | Agree | Agree a lot |
| --- | --- | --- | --- | --- | --- |
| With an examination, a doctor can find skin changes that have not developed into cancer yet, but later might do so [79] | (1) ❑ | (2) ❑ | (3) ❑ | (4) ❑ | (5) ❑ |
| By having your skin checked regularly by a doctor, I will feel secure about my health [80] | (1) ❑ | (2) ❑ | (3) ❑ | (4) ❑ | (5) ❑ |
| Regular skin examinations at a doctor will help me obtain a long life [81] | (1) ❑ | (2) ❑ | (3) ❑ | (4) ❑ | (5) ❑ |
| By examining my own skin regularly, it is easier for me to discover changes in the skin before they become more serious [82] | (1) ❑ | (2) ❑ | (3) ❑ | (4) ❑ | (5) ❑ |
| Examining my own skin for changes is a part of normal self-health care [83] | (1) ❑ | (2) ❑ | (3) ❑ | (4) ❑ | (5) ❑ |
| Examining my own skin for changes makes me feel in control of my own health [84] | (1) ❑ | (2) ❑ | (3) ❑ | (4) ❑ | (5) ❑ |

**Value 0 1 2 3 4**

Scale 11 – Perceived efficiency of protection

To which degree do you disagree or agree on the following:

|  | Diasgree a lot | Disagree | Neither disagree or agree | Agree | Agree a lot |
| --- | --- | --- | --- | --- | --- |
| Regular use of sunscreen with at least SPF 15 when I am in the sun, reduces my risk of skin cancer [85] | (1) ❑ | (2) ❑ | (3) ❑ | (4) ❑ | (5) ❑ |
| Protecting my skin against the sun by applying sunscreen will help me look younger for a longer time [86] | (1) ❑ | (2) ❑ | (3) ❑ | (4) ❑ | (5) ❑ |
| Use of sunscreen (at least SPF 15) is an effective way of avoiding sunburns [87] | (1) ❑ | (2) ❑ | (3) ❑ | (4) ❑ | (5) ❑ |
| By wearing sunprotective clothes (long sleeves, long trousers) when I am in the sun, I reduce my risk of skin cancer [88] | (1) ❑ | (2) ❑ | (3) ❑ | (4) ❑ | (5) ❑ |
| By protecting my skin from the sun with a wide-brimmed hat or a cap when I am in the sun, I reduce my risk of sunburns [89] | (1) ❑ | (2) ❑ | (3) ❑ | (4) ❑ | (5) ❑ |
| Wearing sunprotective clothes (e.g. longsleeved blouses and hat) regularly when I am in the sun, will help my skin look younger for a longer period [90] | (1) ❑ | (2) ❑ | (3) ❑ | (4) ❑ | (5) ❑ |
| My skin does not age so fast if I avoid the sun in the middle of the day [91] | (1) ❑ | (2) ❑ | (3) ❑ | (4) ❑ | (5) ❑ |
| I can avoid sunburns by avoiding the midday sun (between 12 and 3 pm) [92] | (1) ❑ | (2) ❑ | (3) ❑ | (4) ❑ | (5) ❑ |
| Staying in the shades when I am outside will reduce my risk of skin cancer [93] | (1) ❑ | (2) ❑ | (3) ❑ | (4) ❑ | (5) ❑ |
| By wearing sunprotective clothes (long sleeves and long trousers) when I am in the sun, I reduce my risk of sunburns [94] | (1) ❑ | (2) ❑ | (3) ❑ | (4) ❑ | (5) ❑ |
| Staying in the shadows when I am outside will reduce my risk of sunburns [95] | (1) ❑ | (2) ❑ | (3) ❑ | (4) ❑ | (5) ❑ |

**Value 0 1 2 3 4**

Scale 12 – Perceived Barriers Skin examination

To which degree do you disagree or agree on the following:

|  | Diasgree a lot | Disagree | Neither disagree or agree | Agree | Agree a lot |
| --- | --- | --- | --- | --- | --- |
| It would be too expensive and time consuming for me to consult a doctor regularly to get my skin examined [96] | (1) ❑ | (2) ❑ | (3) ❑ | (4) ❑ | (5) ❑ |
| I feel uncomfortable and embarrassed about a doctor examining my skin [97] | (1) ❑ | (2) ❑ | (3) ❑ | (4) ❑ | (5) ❑ |
| I become worried when a doctor examines my skin for changes and signs of skin cancer [98] | (1) ❑ | (2) ❑ | (3) ❑ | (4) ❑ | (5) ❑ |
| I would prefer that a doctor examines my skin for changes and signs of skin cancer rather than myself [99] | (1) ❑ | (2) ❑ | (3) ❑ | (4) ❑ | (5) ❑ |
| I become worried when I examine my own skin for changes and signs of skin cancer [100] | (1) ❑ | (2) ❑ | (3) ❑ | (4) ❑ | (5) ❑ |
| I am just not very good at examining my own skinfor changes or signs of skin cancer [101] | (1) ❑ | (2) ❑ | (3) ❑ | (4) ❑ | (5) ❑ |

**Value 0 1 2 3 4**

| To which degree do you disagree or agree on the following: |
| --- |

Scale 13 – Perceived Barriers Sunscreen

For me, using sunscreen is …

|  | Diasgree a lot | Disagree | Neither disagree or agree | Agree | Agree a lot |
| --- | --- | --- | --- | --- | --- |
| Difficult [102] | (1) ❑ | (2) ❑ | (3) ❑ | (4) ❑ | (5) ❑ |
| Uncomfortable [103] | (1) ❑ | (2) ❑ | (3) ❑ | (4) ❑ | (5) ❑ |
| Expensive [104] | (1) ❑ | (2) ❑ | (3) ❑ | (4) ❑ | (5) ❑ |
| Something I do not like to do [105] | (1) ❑ | (2) ❑ | (3) ❑ | (4) ❑ | (5) ❑ |
| Disturbing for work or leisure time activity [106] | (1) ❑ | (2) ❑ | (3) ❑ | (4) ❑ | (5) ❑ |
| A part of my daily routine [107] | (1) ❑ | (2) ❑ | (3) ❑ | (4) ❑ | (5) ❑ |
| Something that keeps me from getting the tant hat I want [108] | (1) ❑ | (2) ❑ | (3) ❑ | (4) ❑ | (5) ❑ |

**Value 0 1 2 3 4**

| To which degree do you disagree or agree on the following: |
| --- |

Scale 14 – Perceived Barriers Clothing

For me, wearing sunprotective clothes (long sleeves and long trousers) is ...

|  | Diasgree a lot | Disagree | Neither disagree or agree | Agree | Agree a lot |
| --- | --- | --- | --- | --- | --- |
| Difficult [109] | (1) ❑ | (2) ❑ | (3) ❑ | (4) ❑ | (5) ❑ |
| Uncomfortable [110] | (1) ❑ | (2) ❑ | (3) ❑ | (4) ❑ | (5) ❑ |
| Embarrassing and awkward [111] | (1) ❑ | (2) ❑ | (3) ❑ | (4) ❑ | (5) ❑ |
| Not cool [112] | (1) ❑ | (2) ❑ | (3) ❑ | (4) ❑ | (5) ❑ |
| Someting I am uncomfortable doing [113] | (1) ❑ | (2) ❑ | (3) ❑ | (4) ❑ | (5) ❑ |
| Disturbing for work or leisure time activity [114] | (1) ❑ | (2) ❑ | (3) ❑ | (4) ❑ | (5) ❑ |
| A part of my daily routine [115] | (1) ❑ | (2) ❑ | (3) ❑ | (4) ❑ | (5) ❑ |
| Something that keeps me from getting the tant hat I want [116] | (1) ❑ | (2) ❑ | (3) ❑ | (4) ❑ | (5) ❑ |

**Value 0 1 2 3 4**

| To which degree do you disagree or agree on the following: |
| --- |

Scale 23 – Perceived Barriers Hat

For me, wearing a hat as sunprotection is …

|  | Diasgree a lot | Disagree | Neither disagree or agree | Agree | Agree a lot |
| --- | --- | --- | --- | --- | --- |
| Difficult [117] | (1) ❑ | (2) ❑ | (3) ❑ | (4) ❑ | (5) ❑ |
| Uncomfortable [118] | (1) ❑ | (2) ❑ | (3) ❑ | (4) ❑ | (5) ❑ |
| Embarrassing and awkward [119] | (1) ❑ | (2) ❑ | (3) ❑ | (4) ❑ | (5) ❑ |
| Not cool [120] | (1) ❑ | (2) ❑ | (3) ❑ | (4) ❑ | (5) ❑ |
| Someting I am uncomfortable doing [121] | (1) ❑ | (2) ❑ | (3) ❑ | (4) ❑ | (5) ❑ |
| Disturbing for work or leisure time activity [122] | (1) ❑ | (2) ❑ | (3) ❑ | (4) ❑ | (5) ❑ |
| A part of my daily routine [123] | (1) ❑ | (2) ❑ | (3) ❑ | (4) ❑ | (5) ❑ |
| Something that keeps me from getting the tant hat I want [124] | (1) ❑ | (2) ❑ | (3) ❑ | (4) ❑ | (5) ❑ |

**Value 0 1 2 3 4**

| To which degree do you disagree or agree on the following: |
| --- |

Scale15 - Perceived Barriers avoiding the sun between 12 and 3 pm

For me, avoiding the sun between 12 and 3 pm is …

|  | Diasgree a lot | Disagree | Neither disagree or agree | Agree | Agree a lot |
| --- | --- | --- | --- | --- | --- |
| Difficult [125] | (1) ❑ | (2) ❑ | (3) ❑ | (4) ❑ | (5) ❑ |
| Inconvenient [126] | (1) ❑ | (2) ❑ | (3) ❑ | (4) ❑ | (5) ❑ |
| Something that suits me well [127] | (1) ❑ | (2) ❑ | (3) ❑ | (4) ❑ | (5) ❑ |
| Disturbing for work or leisure time activity [128] | (1) ❑ | (2) ❑ | (3) ❑ | (4) ❑ | (5) ❑ |
| A part of my daily routine [129] | (1) ❑ | (2) ❑ | (3) ❑ | (4) ❑ | (5) ❑ |
| Something that keeps me from getting the tant hat I want [130] | (1) ❑ | (2) ❑ | (3) ❑ | (4) ❑ | (5) ❑ |

**Value 0 1 2 3 4**

| To which degree do you disagree or agree on the following: |
| --- |

Scale 16 – Severity Melanoma

|  | Diasgree a lot | Disagree | Neither disagree or agree | Agree | Agree a lot |
| --- | --- | --- | --- | --- | --- |
| I think melanoma is easy to cure [131] | (1) ❑ | (2) ❑ | (3) ❑ | (4) ❑ | (5) ❑ |
| I think melanoma can have very serious consequences [132] | (1) ❑ | (2) ❑ | (3) ❑ | (4) ❑ | (5) ❑ |
| If I get melanoma, it is a large health risk for me [133] | (1) ❑ | (2) ❑ | (3) ❑ | (4) ❑ | (5) ❑ |

**Value 0 1 2 3 4**

scale16 = sum (of MMnemkur MMserius MMriskme)

| Below is a list of risk factors that potentially can affect human health.  For every risk factor, please mark whether you think it poses a high or low risk to human health. |
| --- |

Scale 17 – General Risk Perception

|  | Very high risk | High risk | Normal risk | Low risk | Very low risk |
| --- | --- | --- | --- | --- | --- |
| Tobacco [134] | (1) ❑ | (2) ❑ | (3) ❑ | (4) ❑ | (5) ❑ |
| Obesity [135] | (1) ❑ | (2) ❑ | (3) ❑ | (4) ❑ | (5) ❑ |
| High blood pressure [136] | (1) ❑ | (2) ❑ | (3) ❑ | (4) ❑ | (5) ❑ |
| Low fruit and vegetable intake [137] | (1) ❑ | (2) ❑ | (3) ❑ | (4) ❑ | (5) ❑ |
| High alcohol use [138] | (1) ❑ | (2) ❑ | (3) ❑ | (4) ❑ | (5) ❑ |
| Exposure to UV [139] | (1) ❑ | (2) ❑ | (3) ❑ | (4) ❑ | (5) ❑ |

**Value 0 1 2 3 4**

scale17 = sum (of risktoba riskweig riskbloP riskfrui riskalko riskUV)

Skala 18 – Worry about Melanoma

| To which degree do you disagree or agree on the following: |
| --- |

|  | Disagree a lot | Disagree | Neither disagree or agree | Agree | Agree a lot |
| --- | --- | --- | --- | --- | --- |
| The likelihood of developing skin cancer one day worries me [140] | (1) ❑ | (2) ❑ | (3) ❑ | (4) ❑ | (5) ❑ |
| When I hear about an acquaintance or a famous person with skin cancer it makes me think I can get it too [141] | (1) ❑ | (2) ❑ | (3) ❑ | (4) ❑ | (5) ❑ |
| It would be horrible to get skin cancer [142] | (1) ❑ | (2) ❑ | (3) ❑ | (4) ❑ | (5) ❑ |

**Value 4 3 2 1 0**

scale18 = sum (of worry_me worystar canhorro

Compared to others of your gender, age and skin type, what is the likelihood of developing melanoma in the course of your life (or get melanoma again, if you had it)? [143]

(1) ❑ A lot below the average

(2) ❑ Below the average

(3) ❑ Average

(4) ❑ Over the average

(5) ❑ A lot over the average

| **The next questions are about your beliefs in relation to affecting your risk of skin cancer.** |
| --- |

|  | No control | A little control | Some control | Moderate control | A lot of control |
| --- | --- | --- | --- | --- | --- |
| Overall, how much control do you feel you have, about whether you get skin cancer in the future? [144] | (1) ❑ | (2) ❑ | (3) ❑ | (4) ❑ | (5) ❑ |
| Overall, how much control do you feel you have, about whether you discover a birthmark in an eary stage in the future? [145] | (1) ❑ | (2) ❑ | (3) ❑ | (4) ❑ | (5) ❑ |

Scale 19 – Attitude Tanning

What do you think about sunbathing and being tanned? Please mark to which degree you disagree or agree in the following Venligst markér, i hvilken udstrækning du er enig eller uenig i følgende statements:

|  | Disagree a lot | Disagree | Neither disagree or agree | Agree | Agree a lot |
| --- | --- | --- | --- | --- | --- |
| Being tanned is attractive [146] | (1) ❑ | (2) ❑ | (4) ❑ | (5) ❑ | (6) ❑ |
| Being tanned is healthy [147] | (1) ❑ | (2) ❑ | (4) ❑ | (5) ❑ | (6) ❑ |
| Being tanned makes me look healthy[148] | (1) ❑ | (2) ❑ | (4) ❑ | (5) ❑ | (6) ❑ |
| Being tanned protects a person against sunburns and other skin issues [149] | (1) ❑ | (2) ❑ | (4) ❑ | (5) ❑ | (6) ❑ |
| It is worth getting a little bit sunburned to get a tan [150] | (1) ❑ | (2) ❑ | (4) ❑ | (5) ❑ | (6) ❑ |
| I look better when I am tanned [151] | (1) ❑ | (2) ❑ | (4) ❑ | (5) ❑ | (6) ❑ |
| I feel better when I am tanned [152] | (1) ❑ | (2) ❑ | (4) ❑ | (5) ❑ | (6) ❑ |
| The sun feels good on my skin [153] | (1) ❑ | (2) ❑ | (4) ❑ | (5) ❑ | (6) ❑ |
| I need a tan for my job, sport activity or hobby [154] | (1) ❑ | (2) ❑ | (4) ❑ | (5) ❑ | (6) ❑ |

**Value 0 1 2 3 4**

continued

Here follows some statements about sunbathing (i.e. staying in the sun, partially dressed, to get a tan). Please not to which degree you agree or diagree.

|  | Disagree a lot | Disagree | Neither disagree or agree | Agree | Agree a lot |
| --- | --- | --- | --- | --- | --- |
| Most people I know, finds it nice to have a tan [155] | (1) ❑ | (2) ❑ | (3) ❑ | (4) ❑ | (5) ❑ |
| Most people I know like the look of a tan [156] | (1) ❑ | (2) ❑ | (3) ❑ | (4) ❑ | (5) ❑ |
| Most people I know do not care much about their skincolor [157] | (1) ❑ | (2) ❑ | (3) ❑ | (4) ❑ | (5) ❑ |
| Most people I know do not like to lay in the sun [158] | (1) ❑ | (2) ❑ | (3) ❑ | (4) ❑ | (5) ❑ |
| Most people I know do not like to be pale [159] | (1) ❑ | (2) ❑ | (3) ❑ | (4) ❑ | (5) ❑ |
| I find it nice to have a little color [160] | (1) ❑ | (2) ❑ | (3) ❑ | (4) ❑ | (5) ❑ |
| I like the look af a tan [161] | (1) ❑ | (2) ❑ | (3) ❑ | (4) ❑ | (5) ❑ |
| I don’t care much about my skincolor [162] | (1) ❑ | (2) ❑ | (3) ❑ | (4) ❑ | (5) ❑ |
| I don’t like to lay in the sun [163] | (1) ❑ | (2) ❑ | (3) ❑ | (4) ❑ | (5) ❑ |
| I don’t like to be pale [164] | (1) ❑ | (2) ❑ | (3) ❑ | (4) ❑ | (5) ❑ |

**Value 0 1 2 3 4**

Scale 20 - Social influence sun behavior

How do your family and friends behave in the sun?

|  | None | Some | About half | Most | All |
| --- | --- | --- | --- | --- | --- |
| How big a part of your family protect themselves in the sun (use sunscreen, wear clothes, avoid midday UV exposure)? [165] | (1) ❑ | (2) ❑ | (3) ❑ | (4) ❑ | (5) ❑ |
| How big a part of your friends protect themselves in the sun (use sunscreen, wear clothes, avoid midday UV exposure)? [166] | (1) ❑ | (2) ❑ | (3) ❑ | (4) ❑ | (5) ❑ |
| How big a part of your family do you think regularly check themselves for skin changes (themselves or by consulting a doctor)? [167] | (1) ❑ | (2) ❑ | (3) ❑ | (4) ❑ | (5) ❑ |
| How big a part of your friends do you think regularly check themselves for skin changes (themselves or by consulting a doctor)? [168] | (1) ❑ | (2) ❑ | (3) ❑ | (4) ❑ | (5) ❑ |

**Value 4 3 2 1 0**

Scale 21 – Knowledge about UVR penetration

How much do you agree on the following statements?

You are exposed to UV radiation …

|  | Disagree a lot | Disagree | Neither disagree or agree | Agree | Agree a lot |
| --- | --- | --- | --- | --- | --- |
| When you are in the shades [169] | (1) ❑ | (2) ❑ | (3) ❑ | (4) ❑ | (5) ❑ |
| When you are in the sun without sunbathing [170] | (1) ❑ | (2) ❑ | (3) ❑ | (4) ❑ | (5) ❑ |
| When you are in the water [171] | (1) ❑ | (2) ❑ | (3) ❑ | (4) ❑ | (5) ❑ |
| When it is cloudy [172] | (1) ❑ | (2) ❑ | (3) ❑ | (4) ❑ | (5) ❑ |
| When it rains [173] | (1) ❑ | (2) ❑ | (3) ❑ | (4) ❑ | (5) ❑ |

**Value 0 1 2 3 3**

| **The following questions are about UV radiation from the sun.** |
| --- |

How much do you agree on the following statements?

Scale 21 Knowledge about UV types
The following types of UV radiation can give you skin cancer …

|  | Disagree a lot | Disagree | Neither disagree or agree | Agree | Agree a lot |
| --- | --- | --- | --- | --- | --- |
| UV-A [174] | (1) ❑ | (2) ❑ | (3) ❑ | (4) ❑ | (5) ❑ |
| UV-B [175] | (1) ❑ | (2) ❑ | (3) ❑ | (4) ❑ | (5) ❑ |
| UV-C [176] | (1) ❑ | (2) ❑ | (3) ❑ | (4) ❑ | (5) ❑ |

**Value 0 1 2 3 3**

Skala 21 fortsættes efter 22
Scale 22 – Knowledge about UV and risk of Melanoma

To which degree do you think that the following can pose a risk to the development of skin cancer?

|  | To a very large degree | To a large degree | To some degree | To a low degree | Not at all |
| --- | --- | --- | --- | --- | --- |
| Sunbeds [177] | (1) ❑ | (2) ❑ | (3) ❑ | (4) ❑ | (5) ❑ |
| Vacations to places with more sun than Denmark [178] | (1) ❑ | (2) ❑ | (3) ❑ | (4) ❑ | (5) ❑ |
| Sunburns in childhood [179] | (1) ❑ | (2) ❑ | (3) ❑ | (4) ❑ | (5) ❑ |
| Sunburns in adulthood [180] | (1) ❑ | (2) ❑ | (3) ❑ | (4) ❑ | (5) ❑ |
| Being in the sun between 12 and 3 pm without sun protection [181] | (1) ❑ | (2) ❑ | (3) ❑ | (4) ❑ | (5) ❑ |
| Being in the sun between 12 and 3 pm with sun protection [182] | (1) ❑ | (2) ❑ | (3) ❑ | (4) ❑ | (5) ❑ |
| Sunbathing without sunscreen [183] | (1) ❑ | (2) ❑ | (3) ❑ | (4) ❑ | (5) ❑ |
| Sunbathing with sunscreen | (1) ❑ | (2) ❑ | (3) ❑ | (4) ❑ | (5) ❑ |
| Outdoor work [184] | (1) ❑ | (2) ❑ | (3) ❑ | (4) ❑ | (5) ❑ |

**Value 0 1 2 3 3**

| **Here follows some statements about vitamin D.** |
| --- |

Scale 21 Knowledge about UV and Vitamin D

How much do you agree on the following statements?

|  | Disagree a lot | Disagree | Neither disagree or agree | Agree | Agree a lot |
| --- | --- | --- | --- | --- | --- |
| You can produce vitamin D when you are in the shades [185] | (1) ❑ | (2) ❑ | (3) ❑ | (4) ❑ | (5) ❑ |
| You have to be out between 12 and 3 pm to produce vitamin D [186] | (1) ❑ | (2) ❑ | (3) ❑ | (4) ❑ | (5) ❑ |
| You have to sunbathe to produce vitamin D [187] | (1) ❑ | (2) ❑ | (3) ❑ | (4) ❑ | (5) ❑ |
| If you use sunscreen, you don’t produce enough vitamin D [188] | (1) ❑ | (2) ❑ | (3) ❑ | (4) ❑ | (5) ❑ |
| Between 5 and 20 minuts of sunlight a day is sufficient to cover the need of a person with light skin [189] | (1) ❑ | (2) ❑ | (3) ❑ | (4) ❑ | (5) ❑ |
| Between 3 and 4 hours of sunlight a day is necessary to cover the vitamin D need of a person with light skin [190] | (1) ❑ | (2) ❑ | (3) ❑ | (4) ❑ | (5) ❑ |

**Q1 &Q5 Value 0 1 2 3 3**

Q2Q3Q4Q6 **Value 3 3 2 1 0**

If you have any comments, please note them here:

_______________________________________________________________________________________
_______________________________________________________________________________________
_______________________________________________________________________________________
______________________________________________________________________________________
_______________________________________________________________________________________
_______________________________________________________________________________________

|  |
| --- |
